# Identification of IRF-associated molecular subtypes in clear cell renal cell carcinoma to characterize immunological characteristics and guide therapy

**DOI:** 10.3389/fonc.2022.1118472

**Published:** 2023-01-19

**Authors:** Can Chen, Lin-Yuan Chen, Rui-Xia Yang, Jie-Xin Zhang, Peng-Fei Shao, Hua-Guo Xu

**Affiliations:** ^1^ Department of Laboratory Medicine, The First Affiliated Hospital of Nanjing Medical University, Nanjing, Jiangsu, China; ^2^ Branch of National Clinical Research Center for Laboratory Medicine, Nanjing, Jiangsu, China; ^3^ Department of Urology, The First Affiliated Hospital of Nanjing Medical University, Nanjing, Jiangsu, China

**Keywords:** ccRCC, IRF family, tumour microenvironment, t cell exhaustion, immunotherapy, targeted therapy

## Abstract

**Background:**

Recently studies have identified a critical role for interferon regulatory factor (IRF) in modulating tumour immune microenvironment (TME) infiltration and tumorigenesis.

**Methods:**

Based on IRF1-9 expression profiles, we classified all ccRCC samples into three molecular subtypes (clusters A-C) and characterized the prognosis and immune infiltration of these clusters. IRFscore constructed by principal component analysis was performed to quantify IRF-related subtypes in individual patients.

**Results:**

We proved that IRFscore predicted multiple patient characteristics, with high IRFscore group having poorer prognosis, suppressed TME, increased T-cell exhaustion, increased TMB and greater sensitivity to anti- PD-1/CTLA-4 therapies. Furthermore, analysis of metastatic ccRCC (mccRCC) molecular subtypes and drug sensitivity proved that low IRFscore was more sensitive to targeted therapies. Moreover, IRFscore grouping can be well matched to the immunological and molecular typing of ccRCC. qRT-PCR showed differential expression of IRFs in different cell lines.

**Conclusions:**

Evaluating IRF-related molecular subtypes in individual ccRCC patients not only facilitates our understanding of tumour immune infiltration, but also provides more effective clinical ideas for personalised treatment.

## Introduction

As the most common pathological subtype of kidney cancer, clear cell renal cell carcinoma (ccRCC) is the least malignant but has a high metastatic rate of up to 60% (1). Patients with advanced metastatic kidney cancer are mostly treated with drug therapy, including targeted therapy and immunotherapy (2, 3). Targeted therapies specifically target certain mutated genes or abnormal proteins, which cause less damage to normal cells (4, 5). Some immunotherapeutic drugs are widely used and achieve significant efficacy (2). Actually, researchers found that immunotherapeutic drugs combined with targeted drugs were more effective than monotherapy, which represents a gradual shift in treatment options for kidney cancer towards targeted combination immunotherapy (6).

Interferon regulatory factors (IRFs), can regulate interferons transcriptional modification to fight pathogenic infections (7). Multiple studies confirmed that IRFs regulate tumour immune activity and tumorigenesis. For example, IRF7 high expression potently induces CD8+ T cell responses and strengthens host immune surveillance to fight viral infection and restrict tumour metastasis (8); IRF9 effectively prevents CD8+ T cell exhaustion caused by over-exposure to antigens (9). These results provide a theoretical basis for future studies on tumour immune mechanism and therapeutic applications of IRFs.

In this work, three IRF-related clusters were constructed in ccRCC, and clinical and immune characteristics were assessed between three clusters. Furthermore, we proposed to calculate IRFscore to quantify IRF subtypes in individual patients and proved that IRFscore is highly correlated with patient prognosis, immune infiltration, T-cell exhaustion and treatment. This work will assist clinicians to better understand and differentiate ccRCC immunological and molecular subtypes, and formulate individualised treatment.

## Materials and methods

### Data sources and pre-processing

**Figure S1** illustrated the workflow for this study. We searched and downloaded ccRCC expression datasets with complete clinical annotation and mutations from The Cancer Genome Atlas (TCGA) and Gene Expression Omnibus (GEO) databases. Two datasets (TCGA-KIRC and GSE36895 datasets) were analysed in this work. For TCGA-KIRC dataset, we obtained gene expression data from UCSC website (https://xenabrowser.net/datapages/) and converted them to kilobase per million values. GSE36895 dataset were downloaded from GEO (http://www.ncbi.nlm.nih.gov/geo/). “Sva” package was performed for correcting batch effects in two datasets (10). Samples lacking complete clinical information and mutation data were excluded. Clinical information was summarised in **Table S1**.

### Cell culture

Human renal tubular epithelial cells (HK-2) and ccRCC cell lines (786-O and Caki-1) were obtained from the Cell Bank of the Chinese Academy of Sciences (Shanghai, China). These cells were cultured in DMEM or RPMI-1640 medium containing 10% fetal bovine serum and 1% streptomycin-penicillin. All cells were incubated in a sterile incubator at 5% CO2 and 37°C.

### RNA isolation and quantitative real-time PCR

TRIzol reagent (Invitrogen, USA) was applied to isolate and extract total RNA from the cells. NanoDrop 2000 spectrophotometer (Thermo Scientific, USA) was applied for evaluating of RNA quantity control and concentration. Reverse Transcription Kit (Takara, China) was applied to reverse transcribe total cellular RNA into cDNA. ABI 7500 real-time fluorescence quantitative PCR instrument was designed for carrying out qRT-PCR process. The cycling threshold (Ct) for each gene was recorded and 2-ΔΔCt method was applied to calculate gene mRNA expression. All experiments were repeated 3 times and procedures were carried out according to reagent instructions. Primer sequences were listed in **Table S2**.

### Unsupervised clustering of IRF1-9

Unsupervised clustering analysis were applied to identify IRF-related molecular subtypes. Consensus clustering algorithm was performed for determining the number of clusters. “ConsensuClusterPlus” package was employed to perform consistency clustering analysis (11). The process was repeated a thousand times to ensure consistency of classification.

### Gene set variance analysis

GSVA is a non-parametric unsupervised analysis method that transforms gene expression matrices into gene set expression matrices to evaluate gene set enrichment results of transcriptome (12). Based on the “c2.cp.kegg.v6.2.symbols” gene set obtained from MSigDB database, GSVA analysis was conducted using “GSVA” package.

### Estimation of immune infiltration

Single sample gene set enrichment analysis (ssGSEA) was performed to assess immune infiltration levels based on immune cell-specific gene expression. The immune gene set file is derived from Charoentong et al (13, 14). ESTIMATE algorithm calculates immune and stroma score to estimate the amount of stroma and immune cells and compute tumour purity (15). CIBERSORT is designed to calculate the composition ratio of the 22 immune cells. 22 immune cell expression data are taken from CIBERSORT website (https://cibersort.stanford.edu/) (16). Considering that CD4 naive T cells was 0 in all ccRCC samples, CIBERSORT algorithm only analysed remaining 21 immune cells.

### Identification of DEGs and functional annotation

“limma” package is applied to filter differentially expressed genes (DEGs) between clusters (17). Genes with adjusted P-value<0.001 were recognized as DEGs. “ClusterProfiler” package is intended for GO (Gene Ontology) and KEGG (Kyoto Encyclopedia of Genes and Genomes) enrichment analysis of DEGs (18).

### Construction of IRFscore

Univariate COX regression screened for prognosis-related DEGs. Principal component analysis (PCA) was performed for constructing IRF gene signature. PC1 and PC2 were used as feature scores to calculate IRFscore for individual samples (19). IRFscore = ∑ (PC1i + PC2i), where i represented DEGs’ expression.

### Validation of the clinical value of IRFscore

The TCGA-KIRP and TCGA-KICH cohorts were used to validate the clinical performance of the IRFscore. Information on both queues can be downloaded from the online website (https://portal.gdc.cancer.gov/).

### IPS analysis

The four different immunophenotypic scores (antigen-presenting, effector, suppressor, checkpoint) are calculated separately by immunophenoscore (IPS), IPS z-score is the integration of the four, and the higher the IPS z-score, the more immunogenic the sample (20). IPS was obtained from The Cancer Immunome Atlas (https://tcia.at/home).

### Drug sensitivity analysis

GDSC (https://www.cancerrxgene.org/) database contains massive genomic data on tumour therapeutics and drug sensitivity data (21). We predicted the response of ccRCC patients to five chemotherapeutic agents, including sunitinib, sorafenib, nilotinib, temsirolimus and pazopanib. “pRRophetic” package was performed for quantifying the half maximal inhibitory concentration (IC50).

### Statistics analysis

Protein-protein interaction (PPI) network maps between IRFs was obtained from STRING database (22). Wilcoxon rank sum test was designed to comparative analysis of two groups, Kruskal-Wallis and one-way ANOVA was designed to calculate differences between three and more groups. Spearman correlation analysis was designed to determine correlation coefficient. Kaplan-Meier and log-rank test were performed for plotting survival curves and calculating statistical differences. Multivariate COX regression analysis was conducted to detect independent prognostic factors. “maftools” package was conducted to describe mutations. Statistical analyses were all two-sided and P<0.05 was considered statistically different. All data were analysed by R software (version 4.1.1).

## Results

### Expression pattern and clinical relevance of IRFs in ccRCC

**Figure 1:**
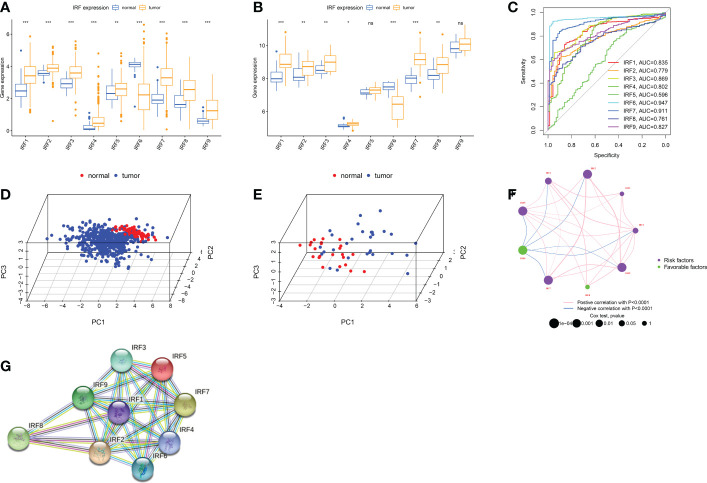
Landscape of IRFs expression in ccRCC. **(A-B)** Boxplot of IRFs expression in ccRCC and normal tissues from TCGA database **(A)** and GSE36895 **(B)**. **(C)** ROC curves demonstrate IRF family ability to differentiate between tumour and normal tissue. **(D-E)** Principal component analysis for the expression profiles of IRFs to distinguish tumours from normal samples in TCGA database **(D)** and GSE36895 **(E)**. **(F)** The interaction between IRFs in ccRCC. **(G)** The PPI network of IRFs. ns, not significant; *p < 0.05; **p < 0.01; ***p < 0.001.

IRF1-9 were included in this work. First, we analysed mRNA expression levels of IRFs in TCGA and GSE36895 cohort, respectively. IRFs were severely imbalanced in expression and the results of both databases remained largely consistent (**Figures 1A, B**). All genes were up-regulated in ccRCC except IRF6. ROC and PCA analysis indicated that IRFs can distinguish well between ccRCC and normal samples (**Figures 1C-E**). We then used two databases, CTPAC and HPA, to compare differential protein expression. CTPAC database results were consistent with the above database (**Figure S2A**). **Figure S2B** illustrated that in HPA database, IRF1, IRF3, IRF7-9 were upregulated in tumour, while the opposite is true for IRF6. IRF2 was highly expressed in both tissues. IRF4 and IRF5 were low or undetectable in both tissues. Furthermore, we observed that IRFs were highly correlated in expression (**Figure 1F**) and interacted with each other in PPI network (**Figure 1G**).

**Figure 2:**
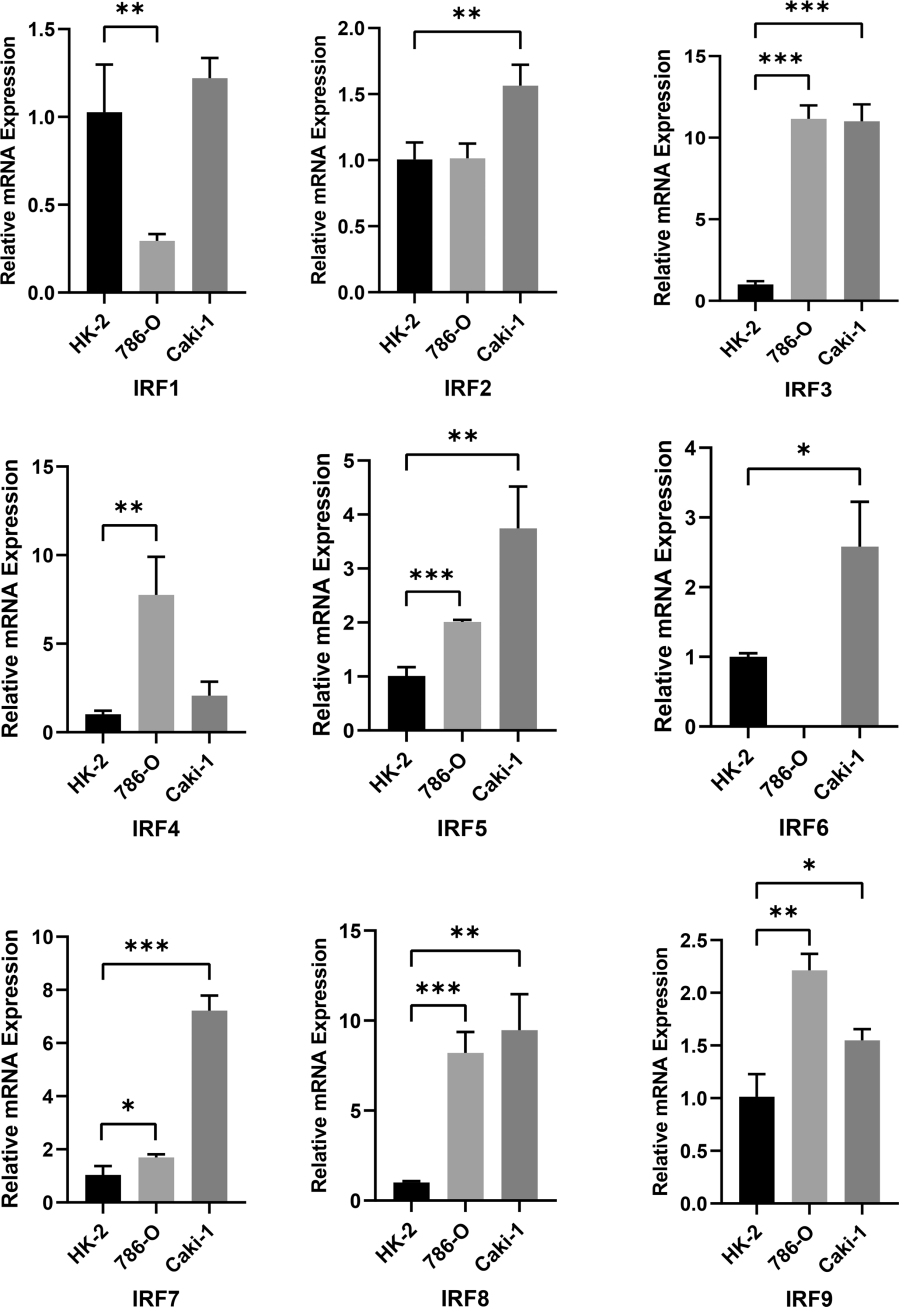
RT-PCR analysis of IRF1-9 expression levels in 786-O, Caki-1 and HK-2 cells. *P < 0.05, **P < 0.01, ***P < 0.001.

To validate IRFs mRNA expression, we performed qRT-PCR analysis in three cell lines. Most IRFs were more highly expressed in tumour cells (**Figure 2**), which is generally consistent with the results above. Furthermore, we noted that IRFs were expressed with cell specificity in different cells (**Figure S2C**).

We then discussed clinical relevance of IRFs. We found that most IRFs were correlated with prognosis (**Figure 1F** and **S2D**). IRF6 exhibited a tumour suppressive profile and its expression was positively correlated with prognosis. In contrast, the higher the expression of other IRFs, the worse the prognosis of patients.

### Identification of IRF-related subtypes in ccRCC

**Figure 3:**
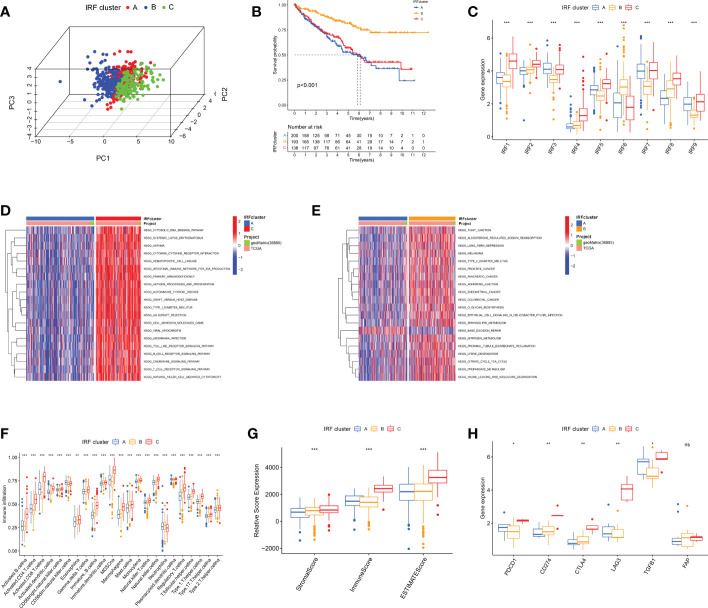
The IRF-related molecular subtypes in ccRCC and biological and immune characteristic of each pattern. **(A)** PCA for the transcriptome profiles of three IRF clusters. **(B)** Survival analyses of three IRF clusters. **(C)** The expression of IRF1-9 in three IRF clusters. **(D-E)** GSVA enrichment analysis showing the activation states of biological pathways in distinct clusters. **(F)** The abundance of each TME infiltrating cell in three clusters. **(G)** Box plot indicated the correlation between IRF clusters and immune scores, stromal scores and estimate scores. **(H)** The expression of most immune checkpoints among distinct IRF clusters. ns, not significant; *p < 0.05; **p < 0.01; ***p < 0.001.

Using an unsupervised clustering approach, we classified ccRCC patients into different subtypes. We ultimately identified three IRF-associated molecular subtypes, termed IRF Cluster A-C (**Figure 3A** and **S3A-C**). Heat maps illustrated the relationship between three subtypes and clinicopathological features (**Figure S3D**). Prognostic analysis pointed to a much higher survival advantage for cluster B (**Figure 3B**). By analysing IRF expression profiles, we observed higher expression of protective factors (IRF6) in cluster B, while the opposite was true for risk factors (e.g. IRF3 and IRF7) (**Figure 3C**). This laterally explained why cluster B had a better prognosis.

### Immune characteristics of different IRF-related subtypes

GSVA analysis was performed to characterise different biological properties. Multiple immune activation-related pathways, including T and B cell receptor signalling pathways accumulated in cluster C (**Figures 3D, E** and **S3E**). Cluster B enriched for some matrix activation pathways, whereas cluster A was mainly associated with immunosuppression and base excision repair. We then proceeded to analyse TME immune infiltration. First, we evaluated 23 immune cell infiltrations using ssGSEA, and almost all immune cells were heavily infiltrated in cluster C (**Figure 3F**). We then ran ESTIMATE algorithm to calculate stromal and immune cell content. Apparently, cluster C had much higher immune and stromal scores, signifying that cluster C had lowest tumour purity (**Figure 3G**). However, no matching survival advantage was found for cluster C with this immune profile. Therefore, we counted the relative proportions of cell subpopulations *via* CIBERSORT. CD8+ T cells and M2 macrophages were more predominant (**Figures S3F-G**). Typically, the higher the expression of CD8+ T cells, the more positive the prognosis (23). Interestingly, we observed the greatest proportion of CD8+ T cells in cluster C and the lowest in cluster B, which is opposite to the prognosis. Researches have revealed that CD8+ T cells are exhausted in ccRCC and secrete numerous immune checkpoints, including PD-1 and CTLA-4. At this point, the higher the intensity of CD8+ T cell infiltration, the worse the prognosis of ccRCC (24). Here, we analysed T cell exhaustion-related immune checkpoint expression. Most checkpoints were highest in cluster C (**Figure 3H**). Combining with previous studies, we speculated IRFs may regulate T-cell exhaustion.

### Comprehensive analysis of IRFs-related DEGs

**Figure 4:**
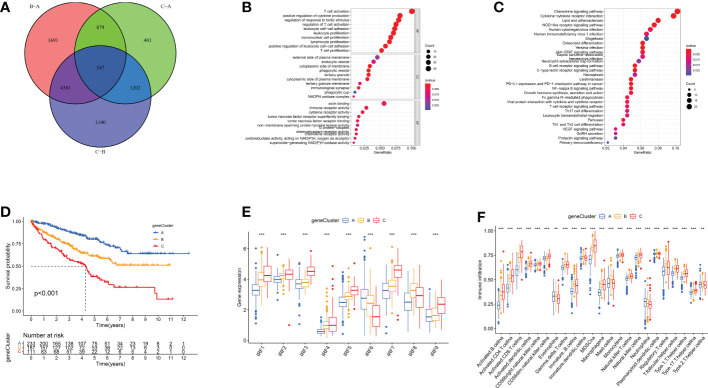
IRF gene clusters in ccRCC and biological characteristics of each gene cluster. **(A)** 547 IRF-associated DEGs shown in venn diagram. **(B-C)** GO **(B)** and KEGG **(C)** enrichment analysis on these DEGs. **(D)** Survival analyses of three IRF gene clusters. **(E)** The expression of IRFs in three gene clusters. **(F)** The abundance of each TME infiltrating cell in three gene clusters. **p < 0.01; ***p < 0.001.

To further characterise biological functions of IRF-related subtypes, we filtered 547 DEGs from three subtypes and performed functional enrichment analysis (**Figure 4A**). These DEGs participated in many immune cell activation and proliferation-related pathways (**Figures 4B, C**). This implied that IRF-associated DEGs are actively engaged in immune processes and modulating immune infiltration. Subsequently, univariate COX regression analysis was performed to identify 426 prognosis-related DEGs (**Table S3**). Similarly, we ran unsupervised cluster analysis on 426 DEGs and identified three gene clusters, termed IRF gene Cluster A-C (**Figures S4A-D**). Similarly, we compared clinicopathological characteristics and immune infiltration between different gene clusters and found that gene cluster A had superior prognostic prospects (p<0.001, **Figures 4D** and **S4E**). Except for IRF6 and IRF8, the remaining risk genes were expressed in gene clusters in the order C, B and A (**Figure 4E**). CD8+ T cells and MDSC had lowest infiltration intensity in Cluster A (**Figure 4F**). This accounted for the greatest survival advantage of gene cluster A. Overall, the concordance of prognostic and immune infiltration characteristics among gene clusters justified this classification.

### Establishment of IRF gene signature and its clinical characteristics

**Figure 5:**
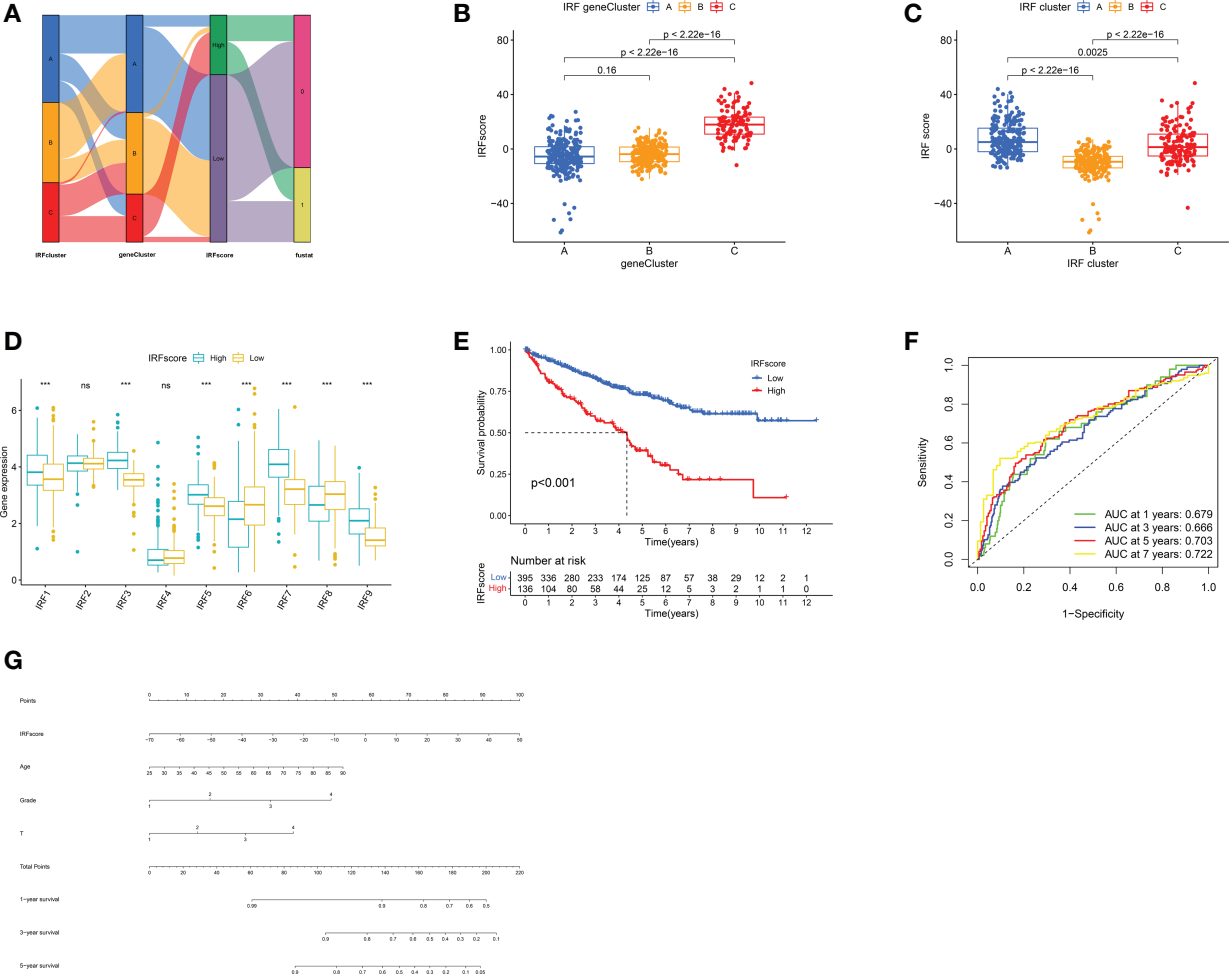
Construction of IRF signatures. **(A)** Alluvial diagram showing the changes of IRF cluster, gene cluster, IRFscore and patient survival status. **(B-C)** Differences in IRFscore among three gene clusters **(B)** and IRF clusters **(C)**. **(D)** The expression of IRF1-9 in two IRFscore groups. **(E)** Kaplan-Meier survival analysis for two IRFscore groups. **(F)** ROCs for 1-, 3-, 5-, and 7-year survival time based on IRFscore. **(G)** Nomograms incorporating IRFscore and clinical characteristics for predicting patient 1-, 3-, 5-year survival. ns, not significant; ***p < 0.001.

PCA analysis was conducted on 426 DEGs and IRFscore were calculated to accurately quantify individual IRF-related molecular subtypes. The samples were divided into high and low IRFscore groups following the threshold values determined by “survminer” package. **Figures 5A-C** exhibited the variation in attributes of individual patients in different clusters. **Figure 5D** demonstrated IRFs expression profiles in two groups. Prognostic analysis revealed that the higher the IRFscore, the worse the prognosis (p<0.001, **Figure 5E**).

Next, we proceeded with a stratified prognostic analysis by different clinical characteristics. First, we observed a higher proportion of patients with advanced tumours were in high-IRFscore group (p<0.05, **Figure S5A**). Patients with VHL, PBRM1 and BAP1 mutations also had higher IRFscore, although not statistically different (**Figure S5B**). Stratified prognostic analysis revealed that low IRFscore consistently showed marked survival advantages (p<0.05, **Figure S5C**). Multivariate Cox regression analysis proved that IRFscore could be independent prognostic factor (**Table S4**). ROC curves and nomograms demonstrated the performance of IRF scores in predicting patients’ rates at 1, 3, 5 and 7 years (AUC≥0.666, **Figures 5F, G**).

### Further validation of IRFscore’s prognostic performance using two independent cohorts

To gain insight into IRFscore’s prognostic value, we further validated the effectiveness of IRFscore in predicting papillary renal cell carcinoma (KIRP) and kidney chromophobe (KICH) prognosis. Based on previous PCA results obtained from 426 DEGs, IRFscore was re-established and survival analyses were performed. In KICH, the prognosis was significantly better in low IRFscore group, while the opposite was true in KIRP (P<0.05, **Figures S5D, E**). This suggested that IRFs are responsible for renal cancer progression, but for specific efficacy, it depended on cancer type.

### Association between IRFscore and CD8+ T cell exhaustion

**Figure 6:**
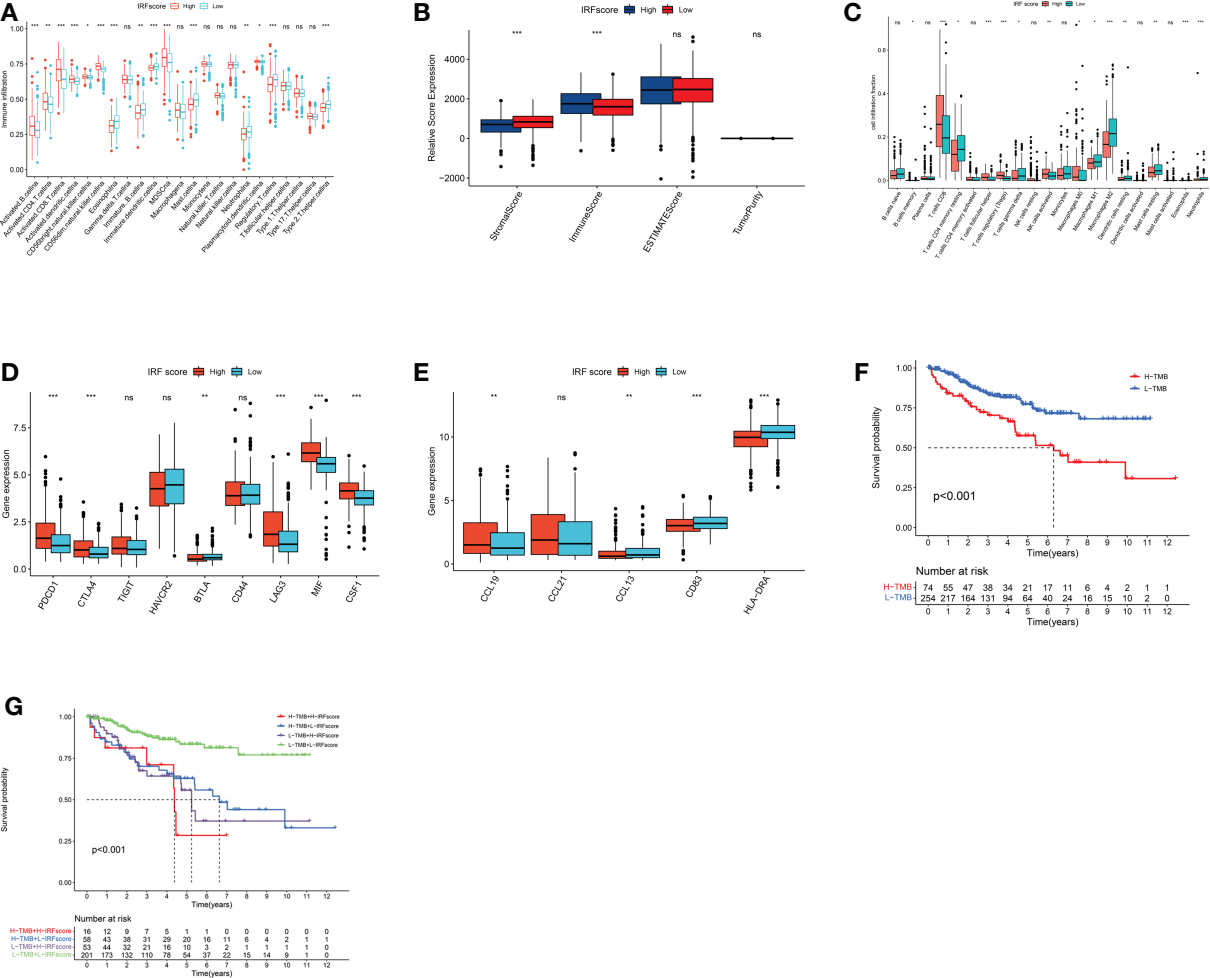
immune characteristics and somatic variants in IRFscore groups. **(A)** The abundance of each TME infiltrating cell in two IRFscore groups. **(B)** The immune scores, stromal scores and estimate score difference in high and low IRFscore groups. **(C)** The relative fraction of each TME-infiltrated cell in two IRFscore groups. **(D)** The differences in the receptors or ligands expressed by exhausted T cells between two IRFscore groups. **(E)** The differences in TLS-related markers between two IRFscore groups. **(F)** Kaplan-Meier survival analysis for two TMB score groups. **(G)** Kaplan-Meier survival analysis for patients stratified by IRFscore and TMB score. ns, not significant; *p < 0.05; **p < 0.01; ***p < 0.001.

To uncover how IRFscore works in regulating TME, we examined immune infiltration in two groups. High IRFscore group had a more significant immune infiltration (**Figures 6A, B**). Furthermore, we found that CD8+ T cells and M2 macrophages accounted for largest proportion in both groups (**Figure 6C**). Therefore, we speculated that these cells probably function primarily in ccRCC progression. Previous studies demonstrated that immune dysregulation occurs in advanced ccRCC (25), when massive exhausted T cells and M2 macrophages are simultaneously enriched in TME and substantial receptor-ligand interactions exist between two cells leading to worse prognosis (26). **Table S5** listed receptors or ligands expressed by two cells. Expression analysis revealed that most co-stimulatory receptors, except for HAVCR2 and BTLA, were significantly overexpressed in high IRFscore group (**Figure 6D**). This suggested that CD8+ T cells in high IRFscore were mostly in exhausted state. However, a matching profile of M2 macrophages was not observed in high IRFscore group (**Figure S6A**). These results indicated that IRFs may not participate in interaction of exhausted T cells with M2 macrophages.

Tertiary lymphoid structures (TLS) are ectopic lymphoid tissues that surround the tumour. The higher the density of its presence, the better the patient’s prognosis (27). In ccRCC, TLS not only occurs significantly less frequently than other cancers, but also becomes dysfunctional (28). Interestingly, when TLS density and mature DCs are increased in ccRCC, a group of patients with high CD8+ T-cell infiltration and good prognosis emerges (29). This contradicted previous findings that CD8+ T cells cause worse prognosis in ccRCC (23). Therefore, scientists assumed that the emergence of TLS and mature DCs could be one reason for reduced T-cell exhaustion (30). We extracted TLS-related markers from published literatures, including three chemokines (CCL19, CCL21 and CXCL13) and two TLS-DC-related markers (HLA-DR and CD83). HLA-DR, CD83 and CCL13 were significantly upregulated in low IRFscore, while only CCL19 was downregulated (**Figure 6E**). Thus, we hypothesized that increased presence of TLS and mature DCs in low IRFscore may enhance ccRCC prognosis by reducing T-cell exhaustion.

### The role of IRFs in TMB and therapy

Many studies proved that the more genetic mutations a tumour has, the more abnormal proteins it produces and the more likely the immune system is to be activated. This implied that tumour mutational burden (TMB) is somewhat predictive of immunotherapy effects (31). Furthermore, TMB can accurately predict multiple targeted and chemotherapeutic drug effects (32). Generally, the higher the TMB, the better the treatment effect. In this work, quantitative analysis and correlation analysis confirmed a positive correlation between IRFscore and TMB (**Figures S6B-C**). Survival analysis proved that lower TMB predicts a good prognosis (p<0.001, **Figure 6F**). We further assessed the synergistic effect of these two scores in prognosis. Stratified survival analysis indicated that TMB and IRFscore did not interfere with each other, with IRFscore showing significant survival differences in two TMB subgroups (p<0.001, **Figure 6G**). This meant that IRFscore could serve as a prognostic indicator independent of TMB.

**Figure 7:**
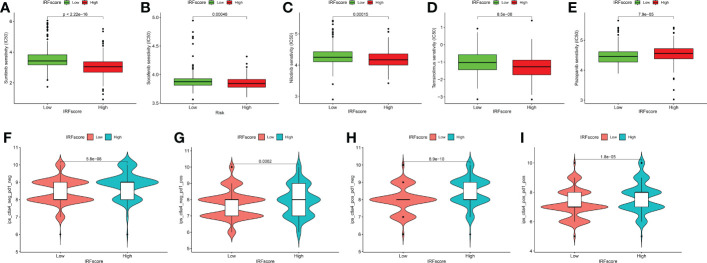
IRFscore in the role of ccRCC clinical therapies. **(A-E)** Box plot showing the sensitivity of patients with high and low IRFscore subgroups to chemotherapy drugs, including sunitinib **(A)**, sorafenib **(B)**, nilotinib **(C)**, temsirolimus **(D)** and pazopanib **(E)**. **(F-I)** The association between IPS and immune checkpoints in ccRCC patients with different IRFscore.

Next, we discussed the performance of IRFscore in predicting targeted therapy efficacy. We compared estimated IC50 of five drugs (**Figures 7A-E**). Except for pazopanib, IC50 levels for remaining drugs were significantly higher in low IRFscore, meaning that low IRFscore was more sensitive to these drugs (p < 0.001). We then investigated the association between IRFscore and immune checkpoint inhibitor (ICI) therapy by IPS. **Figures 7F-I** depicted that four IPS scores were significantly higher in high IRFscore (p<0.001), signifying that higher IRFscore may have higher immunogenic phenotypes and be more sensitive to ICIs. Additionally, the higher the frequency of PBRM1 mutations, the better the outcome of anti-PD-1 treatment was found (26). **Figure S5B** demonstrated that PBRM1 mutations were more frequent in high IRFscore. Above results indicated that low IRFscore group may be more sensitive to targeted therapies, while high IRFscore subgroup were more sensitive to immunotherapy.

### Degree of matching of IRFscore groups to ccRCC immunological and molecular subtypes

Numerous studies indicated that patient response to treatment options can be predicted by different tumour subtypes (33). We therefore sought to understand whether ccRCC-related phenotypes could explain why IRFs influence treatment outcome and analysed the extent to which IRFscore-related subgroups matched these tumour phenotypes. First, combining immune infiltration characteristics (**Figures 6A-C**), we hypothesized that high IRFscore group matched immune-inflamed phenotype, whereas low IRFscore group matched immune-desert phenotype. Generally, immune-inflamed phenotype was more responsive to anti-PD-L1/PD-1 therapies. In contrast, immune-desert phenotypes had no or the weakest response (34). This was consistent with our previous prediction that high IRFscore group was more sensitive to ICI therapies (**Figures 7F-I**).

Generally, targeted therapies are more effective in metastatic ccRCC (mccRCC) than other treatments (2, 3). To accurately predict the effectiveness of tyrosine kinase inhibitor (TKI) therapy in mccRCC, Benoit et al. identified four mccRCC molecular subtypes with different therapeutic effects on sunitinib based on tumour gene mutations, copy number variants (CNV) and methylation status (35). To determine whether this typing was applicable to our work, we collated the distribution of these features across two groups and summarised in **Table S6** and **Figures S6D-L**. We considered that high IRFscore group may correspond to mccRCC 1/4 group, characterised by poor prognosis, low sunitinib sensitivity, increased methylation levels, slightly higher VHL and PBRM1 mutations, higher CNV, highly inflammatory immunosuppressive environment and low stem cell differentiation (**Figures S6D-L**). In contrast, low IRFscore group corresponded to mccRCC 2/3 group, which has the opposite characteristics. Although not all features match exactly, in general we assume that mccRCC subtypes can be applied to describe IRFscore grouping. These results pointed that IRFscore groupings can be well matched to ccRCC immunological and molecular typing, indicating that optimal treatment can be selected according to each patient’s tumour subtype.

## Discussion

Numerous studies highlight the important role of IRFs in regulating host immune responses and tumorigenesis. To date, most studies focused on single IRF and still lack a comprehensive understanding of how entire IRF family integrally regulates cancer development and TME. In our research, we focus on the value of IRF1-9 in modifying ccRCC TME and treatment.

Different ccRCC molecular subtypes and their characteristics have been identified through transcriptome analysis. In our study, we identified three distinct IRF-related subtypes, each with different prognostic and immune characteristics. Combining with previous studies, we hypothesized that cluster C corresponded to immune-inflamed phenotype characterised by massive immune cell infiltration (33). Unlike three immune phenotypes (immune-inflamed, immune-excluded and immune-desert phenotype) that are widely recognised in other tumours (33), David et al. argued that immune-excluded phenotype is rare in ccRCC (25). Thus, combining immune infiltration, we hypothesized that clusters A and B correspond to immune-desert phenotype with low immune infiltration (33). Previous studies demonstrated that CD8+ T cells are exhausted in ccRCC, when the greater the cellular infiltration, the worse the prognosis (28). By analysing the proportion and degree of immune cell infiltration, we observed that Cluster C exhibited significant CD8+ T cell exhaustion characteristics, while Cluster B had relatively few. Comprehensive analysis of prognostic and immunological features plausibly explained why Cluster C had the worst prognosis despite being immunologically activated, while the opposite was true for Cluster B. This meant that immunophenotypic classification of different IRF-related subtypes was reasonable and valid.

According to these DEGs, we classified ccRCC into three distinct gene subtypes, which also have different clinical and immunological profiles. This reaffirmed IRFs’ potential value in predicting survival and shaping different TMEs. Given individual heterogeneity in IRFs expression, we quantified IRF-related molecular subtypes in individual ccRCC patients accurately by IRFscore. Comprehensive analysis suggested that IRFscore not only correlated significantly with clinical features, but also served as an independent prognostic factor. Besides, several mutation-prone genes in ccRCC, including PBRM1, VHL and BAP1, were mutated more frequently in high IRFscore group. It has been well established that these mutations indicate a poor prognosis for patients (36) and PBRM1 mutations substantially increase patient susceptibility to targeted therapies and immunotherapy (37). These results indirectly indicated potential value of IRFscore in predicting patient prognosis, suggesting that IRFs may be critical factors in affecting ccRCC treatment efficacy.

During chronic infection or cancer with continuous antigen stimulation, T cells fail to differentiate effectively into effector and memory T cells, at which point they gradually lose their original effect and become exhausted. This process is accompanied by massive inhibitory receptors (IRS) expression (24). In ccRCC TME, interactions between exhausted CD8+ T cells and M2-like macrophages cause immune dysfunctional circuits (25, 26). However, by analysing two cell infiltrations and corresponding receptor (ligand) expression in IRFscore groups, we did not find significant interactions between two cells. This indicated that IRFs may not regulate this interaction. TLS, existing around the tumour, consists of a B-cell follicular zone with a germinal centre and a T-cell zone with DC-Lamp+ mature DCs (27). During TLS formation, CCL19 and CCL21 recruit immune cells in vicinity of high endothelial vein to form T, B cell areas. CXCL13 recruits lymphoid tissue-inducing factors and initial B cells to inflammatory site and TLS-B cell area, respectively. A reduced risk of death and recurrence of ccRCC has been found when increased frequency of TLS is accompanied by increased CD8+ T-cell infiltration, contradicting the previous belief that CD8+ T cells cause poorer prognosis (29). Therefore, researchers pointed that increased mature TLS in ccRCC may be relevant to reduced T-cell exhaustion (30). In our study, TLS and mature DCs were more frequent in low IRFscore group (high prognosis) and accompanied by reduced CD8+ T-cell exhaustion. We speculated that IRF may improve patient prognosis by influencing TLS frequency.

Targeted therapy is preferred for mccRCC as it is not effective against conventional chemotherapy and radiotherapy (3). Widely recognised kidney cancer targeted agents fall into two categories, TKI and mTOR inhibitors, acting through VHL/HIF/VEGF and PI3K/AKT/mTOR signalling pathways respectively (4). Some TKI drugs, including sorafenib and sunitinib, can slow down neo-angiogenesis by blocking VEGF (38). Temsirolimus and everolimus, as mTOR pathway inhibitors, can block mTOR proteins to exert therapeutic effects (4). Benoit et al. constructed mccRCC-related molecular markers to predict patient response to treatment with sunitinib and identified four different molecular subtypes (mccRCC1-4) (35). Interestingly, we found that high IRFscore matched mccRCC1/4, while low IRFscore matched mccRCC2/3. Therefore, we proposed that IRFscore not only serves as a marker for mccRCC typing, but also predicts targeted therapeutic efficacy. ICIs restore T-cell anti-tumour activity by blocking intra-tumour immunosuppressive signalling (6). PBRM1 mutations, TMB and tumour immunophenotypes influence ICI efficacy to some extent. In this work, we revealed significant associations between IRFscore and PBRM1 mutations, TMB and immunotype and confirmed the predictive value of IRFscore in immunotherapy efficacy.

Due to technical limitations, most conclusions in this paper were based on information from public databases. In future, appropriate clinical cohorts and basic trials will be required to address these issues.

## Conclusion

The IRFscore, constructed based on the transcriptomic expression of the IRF family, has independent prognostic value and can provide accurate survival prediction for ccRCC patients. Furthermore, IRFscore can help us to comprehensively assess the IRF-related immune and molecular subtypes in individual patients and guide more effective individualised clinical treatment.

## Data availability statement

The original contributions presented in the study are included in the article/**Supplementary Material**. Further inquiries can be directed to the corresponding authors.

## Author contributions

All authors are contributors to the article. H-GX, J-XZ and P-FS proposed the idea and participated in research design and execution. CC and L-YC performed bioinformatics analysis, paper writing and experimental manipulation. R-XY modified the manuscript. All authors contributed to the article and approved the submitted version.
