# Supplementary material for: Identification of IRF-associated molecular subtypes in clear cell renal cell carcinoma to characterize immunological characteristics and guide therapy
Source: Front Oncol. 2023 Jan 19;12:1118472. doi: 10.3389/fonc.2022.1118472 (PMC9892447; doi:10.3389/fonc.2022.1118472)
Supplement: Supplementary file 1 [file DataSheet_1.docx]

Supplementary Appendix

This appendix has been provided by the authors to give readers additional information about their work. Supplement to: Identification of IRF-associated molecular subtypes in clear cell renal cell carcinoma to characterize immunological characteristics and guide therapy

**Identification of** **IRF-associated molecular subtypes in clear cell renal cell carcinoma to characterize immunological characteristics and guide therapy**

***Supplemental Materials***

**Table of Contents**

**Table S1** TableS1 Statistics on clinical information of patients in two ccRCC cohorts.

**Table S2** The sequences of primers for IRF family and GAPDH

**Table S3** Univariate Cox regression analysis of 426 IRF-related prognostic DEGs.

**Table S4** Multivariate Cox regression analysis results of clinicopathological characteristics and IRFscore.

**TableS5** The biological interactions between terminally exhausted CD8+ T cell population and the M2-like TAM populations.

**TableS6** Summary of the characteristics of molecular subtypes in the high and low IRFscore groups.

**Figure S1** Overview of this work.

**Figure S2** Protein expression and survival analysis of IRF family.

**Figure S3** biological characteristics in three IRF clusters for ccRCC patients.

**Figure S4** clinical characteristics in three IRF gene clusters for ccRCC patients.

**Figure S5** clinical characteristics of IRFscore in OC patient subtypes and other two kidney tumour.

**Figure S6** IRF-related molecular subtypes in the ccRCC immunological and molecular subtypes.

| **Table S1 Statistics on clinical information of patients in two ccRCC cohorts** | | | | |
| --- | --- | --- | --- | --- |
| **clinical characteristics** | | **TGCA-KIRC cohort（n=519）** | **GSE36895 cohort（n=29)** |  |
| Age | <=65 | 343 | 15 |  |
|  | >65 | 176 | 14 |  |
| Gender | female | 182 | 12 |  |
|  | male | 337 | 17 |  |
| Grade | G1-2 | 238 | 15 |  |
|  | G3-4 | 281 | 14 |  |
| T | T1-2 | 333 | 18 |  |
|  | T3-4 | 186 | 11 |  |
| M | M0 | 411 | 16 |  |
|  | M1-X | 108 | 13 |  |
| N | N0 | 233 | 11 |  |
|  | N1-X | 286 | 18 |  |
| BAP1 | wild type | 448 | 25 |  |
|  | mutant | 71 | 4 |  |
| PBRM1 | wild type | 446 | 16 |  |
|  | mutant | 73 | 13 |  |
| VHL | wild type | 448 | 5 |  |
|  | mutant | 71 | 24 |  |

**Table S2 The sequences of primers for IRF family and GAPDH**

| **Gene** | **Forward Primer** | **Reverse Primer** |
| --- | --- | --- |
| IRF1 | ATGCCCATCACTCGGATGC | CCCTGCTTTGTATCGGCCTG |
| IRF2 | CATGCGGCTAGACATGGGTG | GCTTTCCTGTATGGATTGCCC |
| IRF3 | AGAGGCTCGTGATGGTCAAG | AGGTCCACAGTATTCTCCAGG |
| IRF4 | GCTGATCGACCAGATCGACAG | CGGTTGTAGTCCTGCTTGC |
| IRF5 | GGGCTTCAATGGGTCAACG | GCCTTCGGTGTATTTCCCTG |
| IRF6(1) | CCCCAGGCACCTATACAGC | TCCTTCCCACGGTACTGAAAC |
| IRF6(2) | GGCTGCCGACTCTTCTATGG | CCTGGGAATTTGACCTGCTCC |
| IRF7(1) | GCTGGACGTGACCATCATGTA | GGGCCGTATAGGAACGTGC |
| IRF7(2) | CCCACGCTATACCATCTACCT | GATGTCGTCATAGAGGCTGTTG |
| IRF-8 | ATGTGTGACCGGAATGGTGG | AGTCCTGGATACATGCTACTGTC |
| IRF-9 | GCCCTACAAGGTGTATCAGTTG | TGCTGTCGCTTTGATGGTACT |
| GAPDH | CGGAGTCAACGGATTTGGTCGTAT | AGCCTTCTCCATGGTGGTGAAGAC |

**Table S3 Univariate Cox regression analysis of 426 IRF-related prognostic DEGs**

| **id** | **HR** | **HR.95L** | **HR.95H** | **P-value** |
| --- | --- | --- | --- | --- |
| PIF1 | 5.3897 | 3.7100 | 7.8299 | 9.53E-19 |
| KIF18B | 2.5920 | 2.0919 | 3.2116 | 3.08E-18 |
| HJURP | 2.3983 | 1.9691 | 2.9210 | 3.43E-18 |
| AURKB | 2.0854 | 1.7611 | 2.4693 | 1.56E-17 |
| POFUT2 | 2.7948 | 2.1860 | 3.5731 | 2.42E-16 |
| ADAM8 | 1.8311 | 1.5746 | 2.1294 | 3.97E-15 |
| CHFR | 6.3987 | 4.0192 | 10.1871 | 5.16E-15 |
| TACC3 | 2.5428 | 1.9945 | 3.2417 | 4.98E-14 |
| TMEM150C | 0.5575 | 0.4789 | 0.6491 | 5.09E-14 |
| TCIRG1 | 2.1552 | 1.7637 | 2.6337 | 6.05E-14 |
| DBH-AS1 | 2.0931 | 1.7236 | 2.5419 | 9.16E-14 |
| PTTG1 | 1.7833 | 1.5301 | 2.0783 | 1.31E-13 |
| ADM5 | 3.6319 | 2.5745 | 5.1235 | 2.04E-13 |
| SNHG17 | 2.2867 | 1.8336 | 2.8519 | 2.13E-13 |
| REEP4 | 2.8066 | 2.1284 | 3.7009 | 2.63E-13 |
| MYBL2 | 1.7105 | 1.4813 | 1.9753 | 2.65E-13 |
| UCN | 2.1670 | 1.7560 | 2.6743 | 5.75E-13 |
| ARPC3 | 4.3674 | 2.9172 | 6.5386 | 8.09E-13 |
| EMX2 | 0.6912 | 0.6247 | 0.7648 | 8.23E-13 |
| STAT2 | 2.3365 | 1.8474 | 2.9552 | 1.43E-12 |
| SH3BGRL3 | 2.0625 | 1.6774 | 2.5359 | 6.60E-12 |
| NAT9 | 2.7961 | 2.0831 | 3.7531 | 7.59E-12 |
| C3orf35 | 10.6471 | 5.4066 | 20.9671 | 7.87E-12 |
| PLCL2 | 0.4363 | 0.3438 | 0.5537 | 8.97E-12 |
| VAMP1 | 2.1545 | 1.7276 | 2.6869 | 9.63E-12 |
| NCOA4 | 0.5050 | 0.4148 | 0.6148 | 1.01E-11 |
| OTUD7A | 0.0954 | 0.0484 | 0.1877 | 1.04E-11 |
| KCNN4 | 2.1405 | 1.7164 | 2.6695 | 1.43E-11 |
| TFAP2E | 11.5628 | 5.6642 | 23.6039 | 1.78E-11 |
| NPIPA1 | 4.2077 | 2.7609 | 6.4128 | 2.33E-11 |
| PRSS53 | 2.4205 | 1.8628 | 3.1453 | 3.71E-11 |
| STAC3 | 2.5174 | 1.9118 | 3.3150 | 4.87E-11 |
| IL23A | 3.2206 | 2.2722 | 4.5650 | 4.98E-11 |
| IGFLR1 | 2.1577 | 1.7098 | 2.7230 | 9.30E-11 |
| MICAL1 | 2.0639 | 1.6562 | 2.5720 | 1.09E-10 |
| TMEM44 | 1.9351 | 1.5812 | 2.3682 | 1.49E-10 |
| FNBP1L | 0.5001 | 0.4045 | 0.6182 | 1.52E-10 |
| UNC13D | 2.1700 | 1.7115 | 2.7513 | 1.58E-10 |
| ARHGAP4 | 1.9347 | 1.5805 | 2.3683 | 1.59E-10 |
| RELT | 3.2991 | 2.2883 | 4.7563 | 1.61E-10 |
| FOXD2-AS1 | 2.9250 | 2.1031 | 4.0680 | 1.81E-10 |
| ACVR2A | 0.2660 | 0.1768 | 0.4003 | 2.15E-10 |
| RNF215 | 2.7874 | 2.0269 | 3.8333 | 2.86E-10 |
| KIAA0930 | 2.5094 | 1.8746 | 3.3591 | 6.27E-10 |
| MRGBP | 2.8576 | 2.0474 | 3.9884 | 6.73E-10 |
| MIAT | 1.6365 | 1.3993 | 1.9138 | 6.98E-10 |
| TSPAN7 | 0.6638 | 0.5826 | 0.7562 | 7.33E-10 |
| JAK3 | 1.7526 | 1.4648 | 2.0968 | 8.68E-10 |
| KLF9 | 0.5763 | 0.4832 | 0.6875 | 9.00E-10 |
| RNF166 | 2.4630 | 1.8454 | 3.2873 | 9.36E-10 |
| TRMU | 2.5742 | 1.9016 | 3.4847 | 9.39E-10 |
| CENPT | 2.0310 | 1.6173 | 2.5507 | 1.09E-09 |
| HAPLN3 | 1.7863 | 1.4819 | 2.1533 | 1.15E-09 |
| TRMT1 | 2.1662 | 1.6850 | 2.7847 | 1.63E-09 |
| CNOT6L | 0.3969 | 0.2938 | 0.5362 | 1.73E-09 |
| NME4 | 1.8724 | 1.5264 | 2.2968 | 1.77E-09 |
| CLEC2B | 1.6941 | 1.4263 | 2.0123 | 1.93E-09 |
| SLC40A1 | 0.6469 | 0.5608 | 0.7463 | 2.31E-09 |
| TNFSF14 | 1.5490 | 1.3412 | 1.7890 | 2.60E-09 |
| EZH2 | 2.5569 | 1.8763 | 3.4846 | 2.77E-09 |
| CEP68 | 0.4392 | 0.3348 | 0.5762 | 2.86E-09 |
| ZDHHC18 | 3.7025 | 2.3895 | 5.7370 | 4.67E-09 |
| ADPGK | 3.8545 | 2.4522 | 6.0585 | 4.99E-09 |
| EIF2B4 | 3.6306 | 2.3418 | 5.6287 | 8.24E-09 |
| RCC2 | 2.9520 | 2.0408 | 4.2702 | 9.07E-09 |
| DOK3 | 1.9478 | 1.5516 | 2.4453 | 9.18E-09 |
| MPP3 | 3.1851 | 2.1436 | 4.7328 | 9.84E-09 |
| ARPC1B | 2.0114 | 1.5827 | 2.5561 | 1.10E-08 |
| CASP4 | 2.6175 | 1.8817 | 3.6410 | 1.10E-08 |
| ZNF385A | 1.7487 | 1.4435 | 2.1184 | 1.12E-08 |
| CLEC2D | 1.9046 | 1.5256 | 2.3776 | 1.26E-08 |
| LAT | 3.6287 | 2.3253 | 5.6628 | 1.38E-08 |
| MOAP1 | 0.4560 | 0.3477 | 0.5981 | 1.39E-08 |
| TMEM25 | 0.5223 | 0.4172 | 0.6538 | 1.45E-08 |
| SLC2A6 | 1.9330 | 1.5386 | 2.4285 | 1.51E-08 |
| NPHP3 | 2.8549 | 1.9829 | 4.1105 | 1.69E-08 |
| ADCK5 | 2.1834 | 1.6637 | 2.8656 | 1.81E-08 |
| CMTM7 | 2.0589 | 1.5991 | 2.6510 | 2.14E-08 |
| PLEKHO1 | 1.8322 | 1.4813 | 2.2661 | 2.37E-08 |
| SORL1 | 0.5973 | 0.4983 | 0.7158 | 2.42E-08 |
| PPM1M | 2.5024 | 1.8122 | 3.4554 | 2.53E-08 |
| EDA | 0.4089 | 0.2978 | 0.5615 | 3.26E-08 |
| PARP12 | 2.8103 | 1.9460 | 4.0586 | 3.58E-08 |
| WLS | 0.6497 | 0.5573 | 0.7576 | 3.70E-08 |
| MAP3K8 | 2.0209 | 1.5728 | 2.5966 | 3.77E-08 |
| ZNF836 | 0.2802 | 0.1779 | 0.4415 | 4.09E-08 |
| CNIH2 | 4.4640 | 2.6155 | 7.6188 | 4.13E-08 |
| PPP1R18 | 2.1457 | 1.6325 | 2.8202 | 4.39E-08 |
| SHARPIN | 2.3639 | 1.7351 | 3.2204 | 4.95E-08 |
| E4F1 | 2.5617 | 1.8245 | 3.5968 | 5.55E-08 |
| SPATA6 | 0.3887 | 0.2763 | 0.5468 | 5.75E-08 |
| SLC25A4 | 0.5191 | 0.4094 | 0.6582 | 6.21E-08 |
| RHBDF2 | 2.1888 | 1.6481 | 2.9070 | 6.27E-08 |
| SOCS1 | 1.4570 | 1.2713 | 1.6699 | 6.30E-08 |
| IL15RA | 1.9520 | 1.5281 | 2.4934 | 8.56E-08 |
| SOCS6 | 0.4764 | 0.3630 | 0.6251 | 8.88E-08 |
| PYCARD | 1.5470 | 1.3168 | 1.8175 | 1.11E-07 |
| TAF1C | 1.8540 | 1.4751 | 2.3301 | 1.20E-07 |
| FBXO46 | 2.8423 | 1.9261 | 4.1943 | 1.43E-07 |
| TGFBR3 | 0.5262 | 0.4142 | 0.6685 | 1.47E-07 |
| HEATR9 | 25.9755 | 7.6914 | 87.7248 | 1.56E-07 |
| TRAF5 | 2.1397 | 1.6101 | 2.8434 | 1.58E-07 |
| USP51 | 0.4115 | 0.2948 | 0.5744 | 1.81E-07 |
| CSF1 | 1.9117 | 1.4976 | 2.4403 | 1.97E-07 |
| MANSC1 | 0.5346 | 0.4220 | 0.6774 | 2.14E-07 |
| ACVR2B | 0.2436 | 0.1426 | 0.4162 | 2.35E-07 |
| CCDC110 | 0.4100 | 0.2915 | 0.5766 | 2.97E-07 |
| NPAT | 0.4083 | 0.2896 | 0.5757 | 3.21E-07 |
| ARL6IP4 | 2.0585 | 1.5606 | 2.7152 | 3.21E-07 |
| IP6K2 | 2.4290 | 1.7269 | 3.4165 | 3.41E-07 |
| C2CD2L | 5.0101 | 2.6953 | 9.3130 | 3.50E-07 |
| CTBP2 | 0.4286 | 0.3088 | 0.5949 | 4.09E-07 |
| KIFC2 | 1.6938 | 1.3811 | 2.0774 | 4.20E-07 |
| LIMD2 | 1.5956 | 1.3307 | 1.9132 | 4.56E-07 |
| GGA3 | 3.2341 | 2.0494 | 5.1036 | 4.59E-07 |
| VTI1A | 0.2220 | 0.1235 | 0.3991 | 4.92E-07 |
| EPOR | 1.7490 | 1.4061 | 2.1754 | 5.12E-07 |
| CREBL2 | 0.5136 | 0.3959 | 0.6661 | 5.14E-07 |
| NUBPL | 0.3477 | 0.2301 | 0.5253 | 5.21E-07 |
| HSH2D | 1.7030 | 1.3831 | 2.0969 | 5.30E-07 |
| CHTF8 | 0.4687 | 0.3484 | 0.6306 | 5.55E-07 |
| NFE2L3 | 1.6062 | 1.3339 | 1.9340 | 5.71E-07 |
| GARNL3 | 0.2536 | 0.1480 | 0.4344 | 5.87E-07 |
| HCST | 1.4705 | 1.2638 | 1.7110 | 6.04E-07 |
| ZNF490 | 0.1154 | 0.0491 | 0.2709 | 7.08E-07 |
| ZNF580 | 1.9524 | 1.4957 | 2.5484 | 8.57E-07 |
| SCNN1D | 1.6985 | 1.3752 | 2.0979 | 8.79E-07 |
| APOL2 | 1.5094 | 1.2807 | 1.7790 | 9.07E-07 |
| IL21R-AS1 | 40.9089 | 9.2950 | 180.0466 | 9.17E-07 |
| APOBEC3D | 1.8504 | 1.4468 | 2.3666 | 9.49E-07 |
| CD7 | 1.4406 | 1.2449 | 1.6670 | 9.53E-07 |
| FMNL1 | 1.7681 | 1.4075 | 2.2210 | 9.72E-07 |
| NUP62 | 4.2438 | 2.3754 | 7.5820 | 1.05E-06 |
| NCF4 | 1.6435 | 1.3461 | 2.0068 | 1.08E-06 |
| RPS6KA4 | 2.5351 | 1.7439 | 3.6853 | 1.10E-06 |
| ABCA7 | 1.8404 | 1.4399 | 2.3524 | 1.11E-06 |
| TNFRSF18 | 1.6995 | 1.3727 | 2.1040 | 1.13E-06 |
| CSNK1E | 2.0524 | 1.5364 | 2.7415 | 1.13E-06 |
| ARHGAP6 | 0.4343 | 0.3104 | 0.6077 | 1.13E-06 |
| ZNF579 | 1.7133 | 1.3785 | 2.1293 | 1.21E-06 |
| CNN2 | 1.7371 | 1.3897 | 2.1715 | 1.24E-06 |
| RHOG | 2.3703 | 1.6718 | 3.3607 | 1.27E-06 |
| DOK1 | 2.3408 | 1.6578 | 3.3052 | 1.35E-06 |
| RHEBL1 | 2.5611 | 1.7488 | 3.7507 | 1.35E-06 |
| LINC00944 | 1.8012 | 1.4185 | 2.2870 | 1.37E-06 |
| ELP4 | 0.4682 | 0.3424 | 0.6401 | 1.98E-06 |
| GLI4 | 1.5766 | 1.3068 | 1.9020 | 1.98E-06 |
| QTRT1 | 1.6627 | 1.3478 | 2.0512 | 2.08E-06 |
| IFI16 | 1.8850 | 1.4507 | 2.4492 | 2.09E-06 |
| ASXL2 | 0.4773 | 0.3517 | 0.6479 | 2.10E-06 |
| ARHGAP33 | 1.6121 | 1.3226 | 1.9649 | 2.25E-06 |
| KALRN | 0.3983 | 0.2719 | 0.5833 | 2.26E-06 |
| CYHR1 | 2.1154 | 1.5507 | 2.8859 | 2.26E-06 |
| MS4A14 | 1.7945 | 1.4077 | 2.2876 | 2.36E-06 |
| VMO1 | 1.5921 | 1.3119 | 1.9322 | 2.50E-06 |
| SPG7 | 2.2273 | 1.5946 | 3.1109 | 2.64E-06 |
| FXYD5 | 1.6629 | 1.3449 | 2.0562 | 2.65E-06 |
| RPL36A | 1.6766 | 1.3510 | 2.0807 | 2.72E-06 |
| AKT1S1 | 2.3324 | 1.6365 | 3.3241 | 2.80E-06 |
| SPPL2B | 1.8236 | 1.4180 | 2.3452 | 2.85E-06 |
| ITGB2-AS1 | 1.5337 | 1.2821 | 1.8346 | 2.90E-06 |
| CCDC88B | 1.6713 | 1.3474 | 2.0731 | 2.97E-06 |
| CKLF | 2.2158 | 1.5868 | 3.0942 | 3.01E-06 |
| ACTR10 | 0.4611 | 0.3326 | 0.6394 | 3.46E-06 |
| CD72 | 1.5455 | 1.2853 | 1.8584 | 3.70E-06 |
| TRMT5 | 0.4251 | 0.2957 | 0.6111 | 3.84E-06 |
| CCDC149 | 0.3493 | 0.2236 | 0.5459 | 3.86E-06 |
| G3BP2 | 0.5113 | 0.3844 | 0.6799 | 3.99E-06 |
| SP140L | 2.4069 | 1.6538 | 3.5029 | 4.48E-06 |
| TRAPPC12 | 2.6727 | 1.7543 | 4.0719 | 4.72E-06 |
| ZNF444 | 2.0230 | 1.4937 | 2.7398 | 5.28E-06 |
| DEF6 | 1.5661 | 1.2909 | 1.9000 | 5.37E-06 |
| MCC | 0.5521 | 0.4274 | 0.7131 | 5.40E-06 |
| INPP5A | 0.4752 | 0.3444 | 0.6556 | 5.87E-06 |
| EXD2 | 0.4399 | 0.3081 | 0.6279 | 6.10E-06 |
| NDUFS4 | 0.4471 | 0.3155 | 0.6338 | 6.12E-06 |
| RGS19 | 1.8829 | 1.4311 | 2.4773 | 6.16E-06 |
| ZFYVE1 | 0.4209 | 0.2886 | 0.6140 | 7.07E-06 |
| ACVR2B-AS1 | 0.3440 | 0.2159 | 0.5480 | 7.07E-06 |
| PTOV1 | 1.9975 | 1.4769 | 2.7017 | 7.09E-06 |
| ISG20 | 1.7139 | 1.3545 | 2.1687 | 7.21E-06 |
| CNOT1 | 0.5968 | 0.4763 | 0.7478 | 7.31E-06 |
| LINC00937 | 6.3973 | 2.8425 | 14.3979 | 7.33E-06 |
| NDRG2 | 0.6475 | 0.5351 | 0.7836 | 8.03E-06 |
| EFHD2 | 1.7617 | 1.3739 | 2.2590 | 8.05E-06 |
| OASL | 1.5050 | 1.2572 | 1.8015 | 8.41E-06 |
| SH3BP4 | 0.6015 | 0.4809 | 0.7523 | 8.42E-06 |
| CYBA | 1.5727 | 1.2882 | 1.9199 | 8.66E-06 |
| NABP1 | 1.7498 | 1.3666 | 2.2405 | 9.16E-06 |
| TYMP | 1.5262 | 1.2657 | 1.8403 | 9.50E-06 |
| RRN3P2 | 2.9817 | 1.8342 | 4.8470 | 1.05E-05 |
| GNA11 | 0.4957 | 0.3627 | 0.6775 | 1.07E-05 |
| SAP30BP | 3.0437 | 1.8535 | 4.9981 | 1.09E-05 |
| KMT2E-AS1 | 1.5143 | 1.2586 | 1.8220 | 1.10E-05 |
| SH2D2A | 1.4431 | 1.2251 | 1.6997 | 1.13E-05 |
| PLCB2 | 1.6110 | 1.2993 | 1.9975 | 1.38E-05 |
| GPSM3 | 1.6099 | 1.2988 | 1.9956 | 1.39E-05 |
| CXorf65 | 2.1097 | 1.5059 | 2.9557 | 1.43E-05 |
| HMGCLL1 | 0.1219 | 0.0470 | 0.3166 | 1.54E-05 |
| MAGI1 | 0.5779 | 0.4506 | 0.7413 | 1.59E-05 |
| FRS3 | 2.2400 | 1.5525 | 3.2319 | 1.62E-05 |
| PQBP1 | 2.0258 | 1.4694 | 2.7929 | 1.64E-05 |
| RGS10 | 1.5379 | 1.2637 | 1.8716 | 1.74E-05 |
| BATF | 1.4030 | 1.2017 | 1.6381 | 1.83E-05 |
| WDR20 | 0.4169 | 0.2790 | 0.6228 | 1.94E-05 |
| BCS1L | 2.1764 | 1.5231 | 3.1099 | 1.95E-05 |
| ACAP1 | 1.6601 | 1.3155 | 2.0949 | 1.95E-05 |
| ANKRD23 | 8.2674 | 3.1263 | 21.8631 | 2.07E-05 |
| ITGA9-AS1 | 0.1501 | 0.0625 | 0.3603 | 2.19E-05 |
| METTL17 | 1.7947 | 1.3688 | 2.3529 | 2.32E-05 |
| DGKA | 1.9117 | 1.4160 | 2.5810 | 2.33E-05 |
| PFN1 | 2.0722 | 1.4764 | 2.9086 | 2.53E-05 |
| ARHGAP9 | 1.5468 | 1.2607 | 1.8978 | 2.92E-05 |
| DAZAP2 | 0.4883 | 0.3482 | 0.6847 | 3.24E-05 |
| ANKS3 | 1.8281 | 1.3751 | 2.4302 | 3.29E-05 |
| FCHO1 | 1.6104 | 1.2858 | 2.0170 | 3.35E-05 |
| KAZN | 0.5144 | 0.3754 | 0.7048 | 3.52E-05 |
| PGRMC2 | 0.4739 | 0.3323 | 0.6756 | 3.68E-05 |
| SEMA6D | 0.5123 | 0.3720 | 0.7057 | 4.24E-05 |
| PHYH | 0.6149 | 0.4869 | 0.7767 | 4.50E-05 |
| TMC8 | 1.5059 | 1.2366 | 1.8338 | 4.64E-05 |
| CROCC | 1.9817 | 1.4257 | 2.7545 | 4.68E-05 |
| TRIM3 | 2.9104 | 1.7393 | 4.8699 | 4.76E-05 |
| API5 | 0.4232 | 0.2794 | 0.6410 | 4.93E-05 |
| IRF5 | 1.7944 | 1.3522 | 2.3812 | 5.11E-05 |
| LNX1 | 0.6074 | 0.4772 | 0.7733 | 5.17E-05 |
| TMCO6 | 2.1399 | 1.4795 | 3.0951 | 5.33E-05 |
| RFNG | 1.9288 | 1.4008 | 2.6558 | 5.68E-05 |
| AP4S1 | 0.4014 | 0.2569 | 0.6270 | 6.04E-05 |
| DCAF7 | 0.5168 | 0.3743 | 0.7135 | 6.04E-05 |
| MAP3K10 | 2.1408 | 1.4757 | 3.1056 | 6.07E-05 |
| PTK2 | 0.4010 | 0.2561 | 0.6279 | 6.48E-05 |
| DDX51 | 2.2040 | 1.4946 | 3.2501 | 6.67E-05 |
| ANKRD46 | 0.4957 | 0.3507 | 0.7007 | 7.06E-05 |
| CSK | 2.0423 | 1.4335 | 2.9097 | 7.69E-05 |
| RRP1 | 1.9816 | 1.4092 | 2.7864 | 8.41E-05 |
| ZAP70 | 1.5570 | 1.2486 | 1.9416 | 8.47E-05 |
| SAV1 | 0.5665 | 0.4266 | 0.7523 | 8.60E-05 |
| PTPN21 | 0.5095 | 0.3638 | 0.7135 | 8.67E-05 |
| DPP9 | 1.7012 | 1.3045 | 2.2185 | 8.77E-05 |
| PARVG | 1.6365 | 1.2786 | 2.0944 | 9.14E-05 |
| ITGAX | 1.4309 | 1.1956 | 1.7125 | 9.26E-05 |
| TECPR2 | 0.4685 | 0.3201 | 0.6857 | 9.54E-05 |
| SAMM50 | 0.4652 | 0.3164 | 0.6838 | 9.87E-05 |
| ARRB2 | 1.7874 | 1.3343 | 2.3944 | 9.89E-05 |
| ANKDD1A | 2.6373 | 1.6179 | 4.2990 | 0.0001 |
| SMC2 | 0.5425 | 0.3985 | 0.7386 | 0.000102 |
| CXXC4 | 0.4675 | 0.3184 | 0.6864 | 0.000104 |
| GMIP | 1.6690 | 1.2882 | 2.1625 | 0.000106 |
| HYI | 1.6508 | 1.2812 | 2.1271 | 0.000106 |
| TJAP1 | 2.2436 | 1.4909 | 3.3765 | 0.000107 |
| TRAF1 | 1.5676 | 1.2485 | 1.9683 | 0.000109 |
| CGRRF1 | 0.5084 | 0.3608 | 0.7163 | 0.00011 |
| LILRB2 | 1.5949 | 1.2587 | 2.0209 | 0.000111 |
| STRN4 | 2.5057 | 1.5723 | 3.9932 | 0.000112 |
| TWF2 | 1.9876 | 1.4026 | 2.8165 | 0.000112 |
| EBI3 | 1.4428 | 1.1979 | 1.7379 | 0.000112 |
| NME8 | 3.8699 | 1.9449 | 7.7002 | 0.000116 |
| DET1 | 0.4448 | 0.2942 | 0.6725 | 0.000123 |
| MTUS1 | 0.6402 | 0.5095 | 0.8044 | 0.000129 |
| ATF6 | 0.5001 | 0.3506 | 0.7135 | 0.000133 |
| UBE3D | 0.2517 | 0.1240 | 0.5111 | 0.000135 |
| PCED1B-AS1 | 1.5122 | 1.2222 | 1.8711 | 0.000141 |
| ZFP91 | 0.5419 | 0.3952 | 0.7430 | 0.000142 |
| WAS | 1.4410 | 1.1922 | 1.7417 | 0.000158 |
| EHBP1L1 | 1.7794 | 1.3195 | 2.3997 | 0.000159 |
| FRYL | 0.4714 | 0.3187 | 0.6973 | 0.000166 |
| FBXO6 | 1.5701 | 1.2412 | 1.9863 | 0.000169 |
| LGALS9 | 1.5063 | 1.2160 | 1.8661 | 0.000177 |
| LAG3 | 1.2491 | 1.1120 | 1.4032 | 0.000178 |
| XAF1 | 1.4711 | 1.2016 | 1.8011 | 0.000185 |
| PCDH1 | 0.6711 | 0.5443 | 0.8274 | 0.000189 |
| APOBEC3G | 1.4518 | 1.1936 | 1.7657 | 0.00019 |
| ZNF566 | 0.3395 | 0.1925 | 0.5987 | 0.00019 |
| SYTL1 | 1.5146 | 1.2178 | 1.8838 | 0.000191 |
| TRIM44 | 0.5164 | 0.3646 | 0.7313 | 0.000198 |
| PCED1B | 1.5237 | 1.2200 | 1.9029 | 0.000204 |
| MYO9B | 1.8834 | 1.3468 | 2.6339 | 0.000216 |
| PGAP1 | 0.4942 | 0.3401 | 0.7182 | 0.00022 |
| RBMXL1 | 0.4937 | 0.3393 | 0.7184 | 0.000226 |
| PSTPIP1 | 1.4677 | 1.1967 | 1.8000 | 0.000229 |
| TTC21A | 2.4036 | 1.5070 | 3.8336 | 0.000231 |
| FCGR2C | 1.4363 | 1.1843 | 1.7418 | 0.000234 |
| NAGK | 2.2174 | 1.4485 | 3.3945 | 0.000247 |
| TBC1D10C | 1.4583 | 1.1912 | 1.7851 | 0.000256 |
| ZNF787 | 1.7505 | 1.2962 | 2.3641 | 0.00026 |
| TTC7B | 0.4488 | 0.2911 | 0.6919 | 0.000286 |
| ACD | 2.0526 | 1.3917 | 3.0274 | 0.000287 |
| DNAJC18 | 0.5072 | 0.3512 | 0.7324 | 0.000294 |
| TUT1 | 1.9739 | 1.3653 | 2.8538 | 0.0003 |
| SPI1 | 1.4090 | 1.1698 | 1.6971 | 0.000303 |
| DUS3L | 2.0004 | 1.3716 | 2.9176 | 0.000317 |
| SLC25A53 | 0.1647 | 0.0617 | 0.4398 | 0.000319 |
| TSSC4 | 1.6344 | 1.2494 | 2.1379 | 0.000337 |
| RASAL3 | 1.5401 | 1.2155 | 1.9513 | 0.000348 |
| MIB2 | 1.8646 | 1.3242 | 2.6256 | 0.00036 |
| FGL2 | 0.7722 | 0.6698 | 0.8901 | 0.000364 |
| SHROOM2 | 0.5799 | 0.4295 | 0.7831 | 0.000377 |
| YWHAB | 0.4955 | 0.3362 | 0.7301 | 0.000385 |
| CALML6 | 5.3538 | 2.1189 | 13.5273 | 0.000388 |
| MTMR9LP | 1.3694 | 1.1505 | 1.6300 | 0.000405 |
| CARD11 | 1.2916 | 1.1205 | 1.4890 | 0.000418 |
| PARP15 | 1.9209 | 1.3333 | 2.7673 | 0.000458 |
| FLT3LG | 2.1200 | 1.3916 | 3.2295 | 0.000468 |
| EXTL2 | 0.5312 | 0.3727 | 0.7571 | 0.000468 |
| RUSC1 | 2.2455 | 1.4238 | 3.5415 | 0.000502 |
| KLHL7 | 0.4720 | 0.3089 | 0.7214 | 0.000523 |
| UNC93B1 | 1.7245 | 1.2665 | 2.3482 | 0.00054 |
| SLC31A1 | 0.6203 | 0.4730 | 0.8136 | 0.000558 |
| CCL22 | 0.5053 | 0.3418 | 0.7470 | 0.000622 |
| LRRC49 | 0.4270 | 0.2618 | 0.6963 | 0.000649 |
| NCOA2 | 0.6175 | 0.4670 | 0.8166 | 0.000721 |
| ZNF683 | 1.4085 | 1.1533 | 1.7201 | 0.000783 |
| IFI35 | 1.6899 | 1.2431 | 2.2973 | 0.000812 |
| MAP4K1 | 1.3954 | 1.1473 | 1.6972 | 0.000853 |
| MYO1F | 1.4728 | 1.1727 | 1.8498 | 0.000868 |
| INO80B | 1.5277 | 1.1884 | 1.9639 | 0.000944 |
| CLK3 | 2.2661 | 1.3946 | 3.6823 | 0.000958 |
| MIER2 | 1.9245 | 1.3045 | 2.8391 | 0.000967 |
| ZNF865 | 1.7530 | 1.2536 | 2.4514 | 0.001035 |
| IL18BP | 1.4694 | 1.1660 | 1.8517 | 0.001107 |
| PTPN7 | 1.3840 | 1.1380 | 1.6831 | 0.001135 |
| RFXANK | 1.7091 | 1.2376 | 2.3603 | 0.001137 |
| ZCCHC14 | 0.6351 | 0.4828 | 0.8353 | 0.001166 |
| UCKL1 | 1.6663 | 1.2236 | 2.2692 | 0.001192 |
| TREML1 | 2.0611 | 1.3284 | 3.1978 | 0.00125 |
| RAC2 | 1.2963 | 1.1070 | 1.5181 | 0.001276 |
| LINC00528 | 2.6827 | 1.4686 | 4.9003 | 0.001326 |
| PTPN6 | 1.5612 | 1.1886 | 2.0506 | 0.001367 |
| PRDM11 | 0.4062 | 0.2339 | 0.7055 | 0.001382 |
| SH3BP1 | 1.5732 | 1.1912 | 2.0777 | 0.001408 |
| MAPK11 | 1.4633 | 1.1574 | 1.8500 | 0.001466 |
| DENND6B | 1.5712 | 1.1876 | 2.0786 | 0.001556 |
| TSPYL2 | 1.3306 | 1.1145 | 1.5886 | 0.001583 |
| HOXA7 | 0.6498 | 0.4967 | 0.8501 | 0.001665 |
| PEX7 | 0.6188 | 0.4585 | 0.8352 | 0.00171 |
| TMEM8B | 0.6341 | 0.4768 | 0.8435 | 0.00175 |
| WDR83 | 1.7170 | 1.2221 | 2.4123 | 0.001833 |
| OTUD7B | 0.4862 | 0.3084 | 0.7665 | 0.001903 |
| TSPAN32 | 2.0764 | 1.3088 | 3.2942 | 0.001916 |
| CCL5 | 1.1919 | 1.0663 | 1.3324 | 0.001999 |
| MYH7B | 2.3972 | 1.3742 | 4.1817 | 0.002074 |
| CARD16 | 1.4331 | 1.1396 | 1.8020 | 0.002082 |
| CORO1A | 1.2916 | 1.0974 | 1.5202 | 0.00209 |
| FAM111A | 1.5412 | 1.1698 | 2.0304 | 0.002105 |
| TRAF3IP2-AS1 | 0.3249 | 0.1582 | 0.6672 | 0.0022 |
| GVINP1 | 0.6015 | 0.4344 | 0.8329 | 0.002209 |
| DMAP1 | 1.7283 | 1.2126 | 2.4632 | 0.002474 |
| INSL3 | 1.5811 | 1.1751 | 2.1273 | 0.002479 |
| C1orf162 | 1.3338 | 1.1053 | 1.6095 | 0.002666 |
| CAPRIN1 | 0.5924 | 0.4207 | 0.8340 | 0.002705 |
| GZMB | 1.2893 | 1.0913 | 1.5233 | 0.002817 |
| IL2RG | 1.2346 | 1.0751 | 1.4177 | 0.002828 |
| PSMB10 | 1.3738 | 1.1153 | 1.6922 | 0.002831 |
| KIAA0895L | 1.3151 | 1.0979 | 1.5751 | 0.00293 |
| LST1 | 1.3446 | 1.1061 | 1.6346 | 0.002961 |
| MIPOL1 | 0.2879 | 0.1258 | 0.6586 | 0.003188 |
| PLEKHB2 | 0.6941 | 0.5444 | 0.8849 | 0.003212 |
| HRAS | 1.4939 | 1.1435 | 1.9517 | 0.003251 |
| CCDC159 | 1.5471 | 1.1563 | 2.0699 | 0.003309 |
| SMIM8 | 0.3410 | 0.1664 | 0.6991 | 0.003309 |
| LOH12CR2 | 0.4669 | 0.2804 | 0.7774 | 0.003409 |
| BAZ1A | 1.6468 | 1.1774 | 2.3034 | 0.003573 |
| LTB | 1.2331 | 1.0707 | 1.4201 | 0.003635 |
| NLRC5 | 1.3688 | 1.1077 | 1.6914 | 0.003652 |
| WDR74 | 1.8974 | 1.2309 | 2.9247 | 0.003721 |
| GPR132 | 1.5426 | 1.1508 | 2.0678 | 0.003735 |
| NSUN3 | 0.4809 | 0.2921 | 0.7920 | 0.004021 |
| ZBTB48 | 1.6479 | 1.1720 | 2.3171 | 0.004069 |
| STAT4 | 1.6206 | 1.1642 | 2.2560 | 0.004229 |
| HMG20B | 1.7563 | 1.1937 | 2.5842 | 0.004254 |
| CDC34 | 1.6572 | 1.1710 | 2.3453 | 0.004362 |
| SHROOM1 | 1.4722 | 1.1268 | 1.9235 | 0.004578 |
| MBNL3 | 0.7355 | 0.5931 | 0.9121 | 0.00515 |
| GZMH | 1.2298 | 1.0624 | 1.4236 | 0.005594 |
| LRRC8B | 0.5742 | 0.3868 | 0.8522 | 0.00589 |
| CCR4 | 0.6601 | 0.4909 | 0.8877 | 0.005989 |
| DHRS4-AS1 | 0.6672 | 0.4992 | 0.8917 | 0.006245 |
| CCDC142 | 1.8269 | 1.1842 | 2.8185 | 0.006443 |
| EPCAM | 0.8542 | 0.7625 | 0.9570 | 0.006557 |
| CMTR2 | 0.6093 | 0.4255 | 0.8726 | 0.006858 |
| IKZF2 | 0.5447 | 0.3480 | 0.8524 | 0.007839 |
| KAT8 | 1.8038 | 1.1586 | 2.8083 | 0.009007 |
| TMEM134 | 1.5049 | 1.1067 | 2.0463 | 0.009147 |
| MST1 | 1.2431 | 1.0550 | 1.4646 | 0.009314 |
| PRR22 | 1.4434 | 1.0934 | 1.9054 | 0.009586 |
| TTLL5 | 0.4762 | 0.2713 | 0.8357 | 0.009727 |
| PIK3CG | 0.7134 | 0.5520 | 0.9219 | 0.009847 |
| ZMYND15 | 1.4975 | 1.1005 | 2.0377 | 0.01019 |
| CACNA2D2 | 0.3346 | 0.1428 | 0.7838 | 0.01171 |
| ASB9 | 0.6878 | 0.5111 | 0.9256 | 0.013499 |
| TMC6 | 1.4982 | 1.0851 | 2.0686 | 0.014043 |
| AVPR1A | 0.8116 | 0.6870 | 0.9589 | 0.014152 |
| POLR2B | 0.6444 | 0.4535 | 0.9157 | 0.014245 |
| XCR1 | 0.6395 | 0.4469 | 0.9150 | 0.014444 |
| AF186192.1 | 0.6108 | 0.4095 | 0.9111 | 0.015677 |
| ITGA4 | 0.7827 | 0.6405 | 0.9565 | 0.016624 |
| GBP4 | 0.8418 | 0.7295 | 0.9713 | 0.018316 |
| SEMA4D | 0.6850 | 0.4997 | 0.9390 | 0.018726 |
| CD37 | 1.2761 | 1.0394 | 1.5667 | 0.019838 |
| MPEG1 | 0.8343 | 0.7162 | 0.9719 | 0.02002 |
| ITGB7 | 0.6831 | 0.4950 | 0.9426 | 0.020345 |
| LYZ | 0.8923 | 0.8099 | 0.9830 | 0.0211 |
| FAM49B | 1.4556 | 1.0560 | 2.0063 | 0.021857 |
| MYO1G | 1.2995 | 1.0383 | 1.6264 | 0.022129 |
| TNRC6C-AS1 | 1.5837 | 1.0659 | 2.3530 | 0.02284 |
| USP30 | 0.6271 | 0.4189 | 0.9387 | 0.02337 |
| ZBTB32 | 2.3521 | 1.1172 | 4.9522 | 0.024343 |
| ATF4 | 1.4107 | 1.0449 | 1.9045 | 0.024634 |
| FBXL8 | 1.3698 | 1.0402 | 1.8039 | 0.025032 |
| CD5L | 0.8028 | 0.6619 | 0.9736 | 0.025593 |
| HES4 | 1.1871 | 1.0197 | 1.3819 | 0.026973 |
| TRIP10 | 1.3289 | 1.0323 | 1.7107 | 0.027357 |
| JAK2 | 0.7174 | 0.5329 | 0.9658 | 0.028562 |
| FAM210B | 0.6578 | 0.4511 | 0.9590 | 0.029447 |
| AKNA | 1.3147 | 1.0254 | 1.6856 | 0.030976 |
| KCNA3 | 0.6101 | 0.3855 | 0.9655 | 0.034858 |
| P2RY13 | 0.8093 | 0.6646 | 0.9855 | 0.035305 |
| TNNI2 | 1.3442 | 1.0146 | 1.7810 | 0.039313 |
| CTSW | 1.1535 | 1.0069 | 1.3215 | 0.039432 |
| C15orf61 | 0.6119 | 0.3828 | 0.9783 | 0.040201 |
| CFL2 | 0.7579 | 0.5813 | 0.9882 | 0.040589 |
| GNAI1 | 0.7817 | 0.6175 | 0.9896 | 0.040651 |
| IRF1 | 1.2137 | 1.0036 | 1.4678 | 0.045865 |
| MAGI2 | 0.6273 | 0.3944 | 0.9977 | 0.048878 |
| WDR13 | 1.2969 | 1.0012 | 1.6798 | 0.048907 |

**Table S4 Multivariate Cox regression analysis results of clinicopathological characteristics and IRFscore**

| **Clinical characteristics** | **HR** | **HR.95L** | **HR.95H** | **pvalue** |
| --- | --- | --- | --- | --- |
| Age | 1.0320 | 1.0170 | 1.0472 | 0.0000 |
| Gender | 1.1106 | 0.7946 | 1.5521 | 0.5393 |
| Stage | 2.1083 | 1.5844 | 2.8055 | 0.0000 |
| Grade | 1.5085 | 1.1899 | 1.9125 | 0.0007 |
| T | 0.7190 | 0.5200 | 0.9941 | 0.0460 |
| M | 0.8626 | 0.6301 | 1.1809 | 0.3563 |
| N | 0.9061 | 0.8144 | 1.0081 | 0.0701 |
| IRFscore | 1.0232 | 1.0121 | 1.0345 | 0.0000 |

**Table S5 The biological interactions between terminally exhausted CD8+ T cell population and the M2-like TAM populations**

| **Terminally exhausted CD8+ T cell** | **M2-like TAMs** | **corresponding effect** |
| --- | --- | --- |
| PDCD1(PD-1) | PD-L1, PDCD1LG2 | T cell inhibitory signaling |
| CTLA-4 | CD80, CD86 | T cell inhibitory signaling |
| TIGIT | PVR(CD155), NECTIN2(CD112) | T cell inhibitory signaling |
| HAVCR2(TIM-3) | LGALS9(Galectin-9) | T cell inhibitory signaling |
| BTLA | TNFRSF14(HVEM) | T cell inhibitory signaling |
| CD44 | SPP1 | T cell inhibitory signaling |
| MIF | CD74 | M2-like polarization |
| CSF1 | CSF1R | M2-like polarization |

**Table S6 Summary of the characteristics of molecular subtypes in the high and low IRFscore groups**

| **Characteristics** | **High IRFscore group** | **Low IRFscore group** |
| --- | --- | --- |
| sunitinib sensitivity (IC50) | Low | High |
| overall survival | Low (37.5%) | High (42.3%) |
| VHL mutations | High (17%) | Low (12%) |
| PBRM1 mutations | High (17%) | Low (15%) |
| methylation status | High | Low |
| MYC expression level | Low | High |
| polycomb stem-cell phenotype | Low | High |
| inflammatory state | High | Low |
| corresponding molecular subtypes | ccrcc1&4 | ccrcc2&3 |


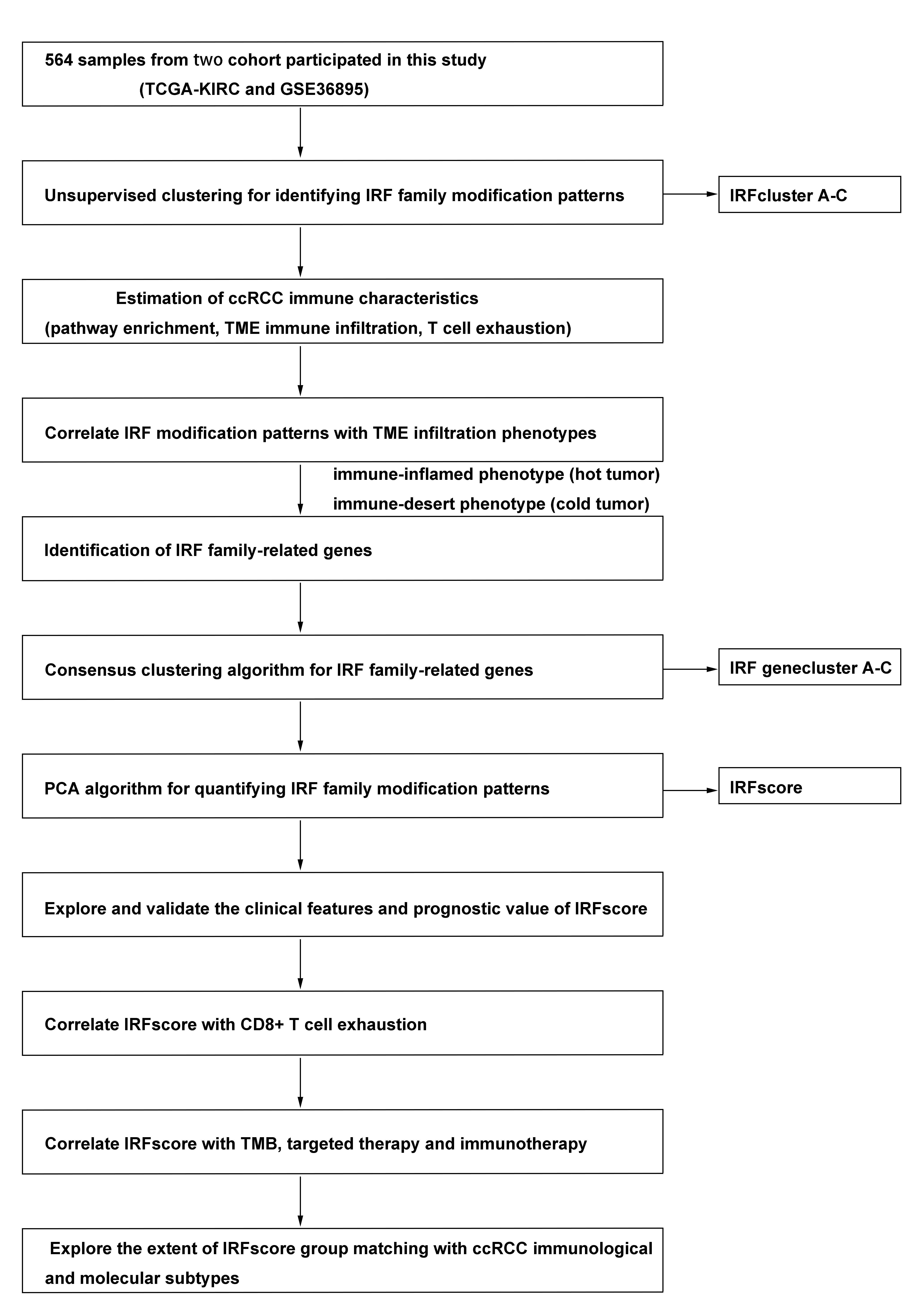


**Figure S1** Overview of this work.


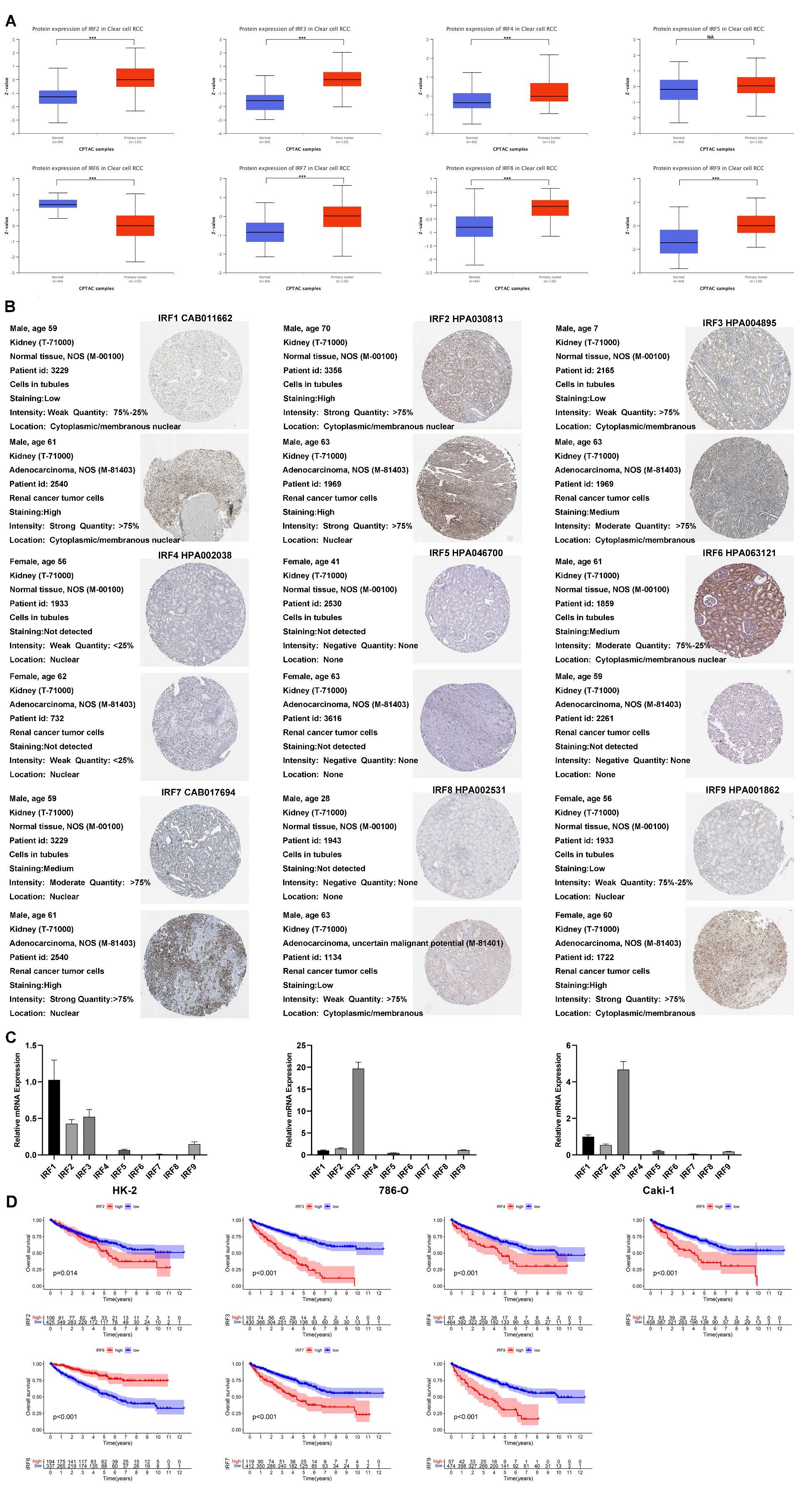


**Figure S2** Protein expression and survival analysis of IRF family. **(A)** Protein expression of IRF2-9 in the tumor and normal tissues in CTPAC database. **(B)** Protein expression of IRF1-9 in tumour and normal tissues in HPA database. **(C)** mRNA expression levels of IRF1-9 in different cell lines, including HK-2, 786-O and Caki-1. **(D)** OS survival curves of IRF1-9.


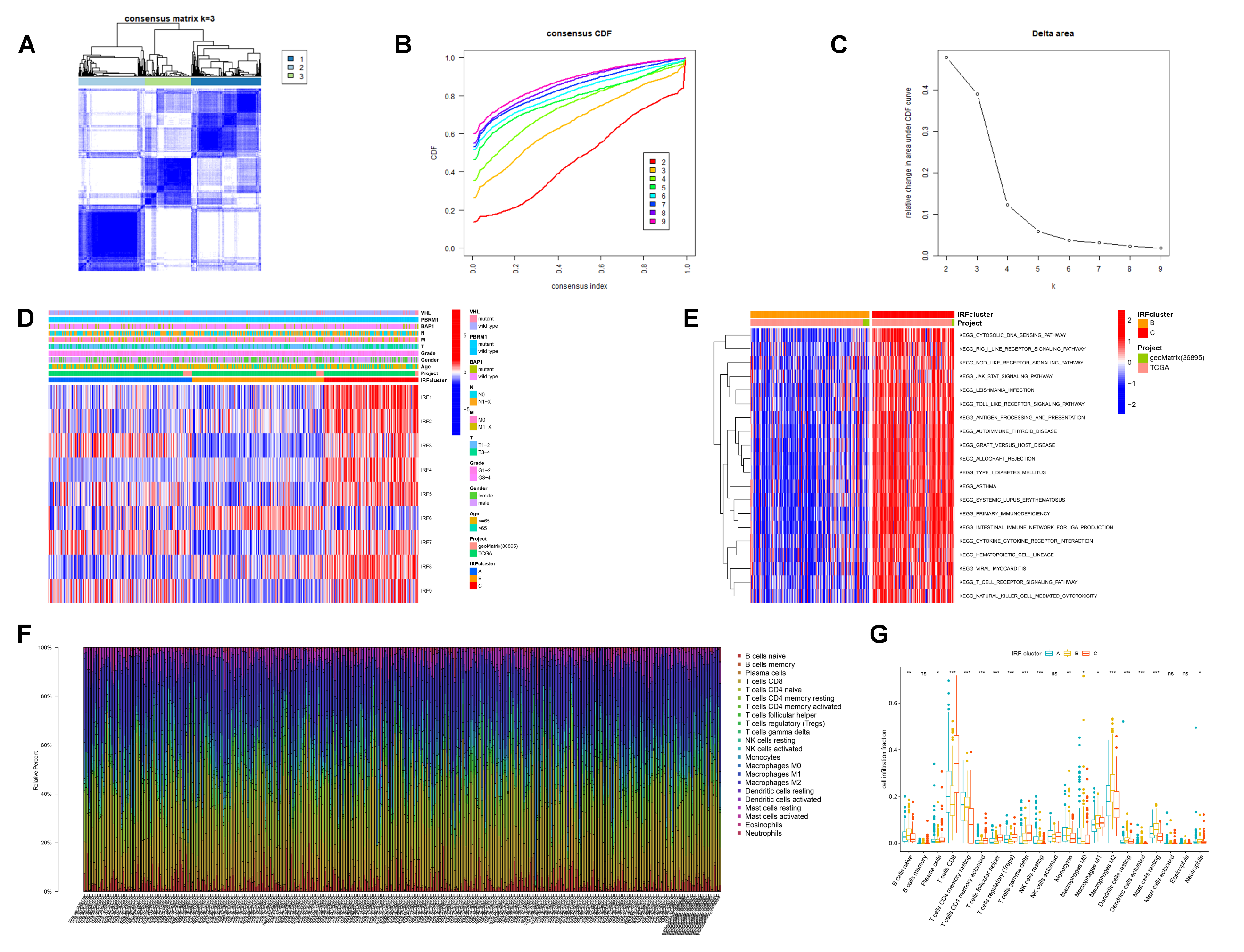


**Figure S3** biological characteristics in three IRF clusters for ccRCC patients. **(A)** Heat map of the consensus matrix for the ccRCC sample at k = 3. **(B)** Cumulative distribution function curves for unsupervised clustering of IRF family, k = 2-9. **(C)** Relative change in area under the CDF curve for unsupervised clustering of IRF family, k = 2-9. **(D)** Heatmap depicted the correlation between IRF clusters and different clinicopathological feature. **(E)** GSVA enrichment analysis showing the activation states of biological pathways in IRF cluster and C. **(F)** The relative fraction of each TME-infiltrated cell in each ccRCC sample. **(G)** The relative fraction of each TME-infiltrated cell in distinct IRF clusters.


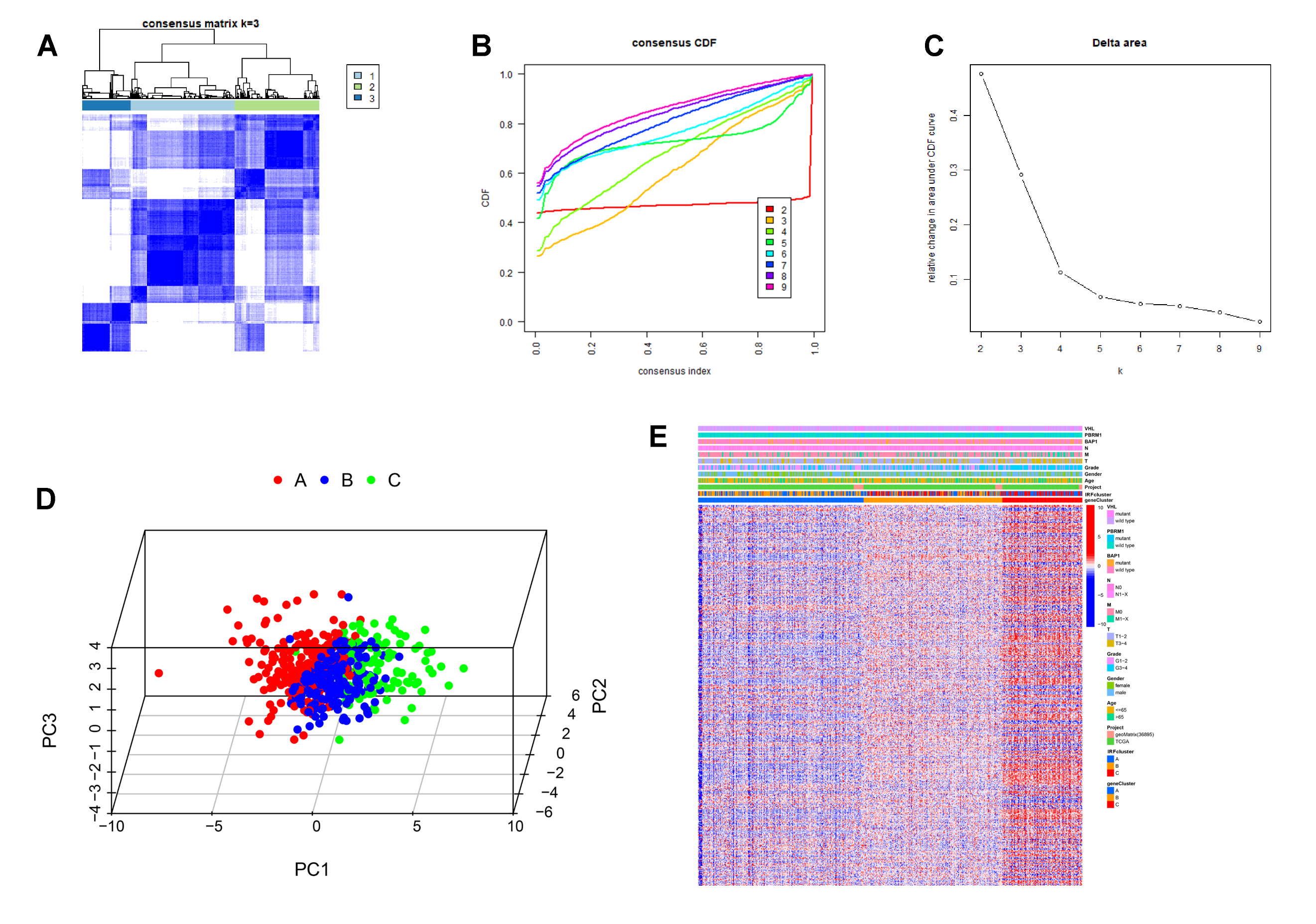


**Figure S4** clinical characteristics in three IRF gene clusters for ccRCC patients. **(A)** Heat map of the consensus matrix for k = 3. **(B)** Cumulative distribution function curves for unsupervised clustering of 426 DEGs, k = 2-9. **(C)** Relative change in area under the CDF curve for unsupervised clustering of 426 DEGs, k = 2-9. **(D)** PCA for the transcriptome profiles of three IRF gene clusters. **(E)** Heatmap depicted the correlation between IRF gene clusters and different clinicopathological features.


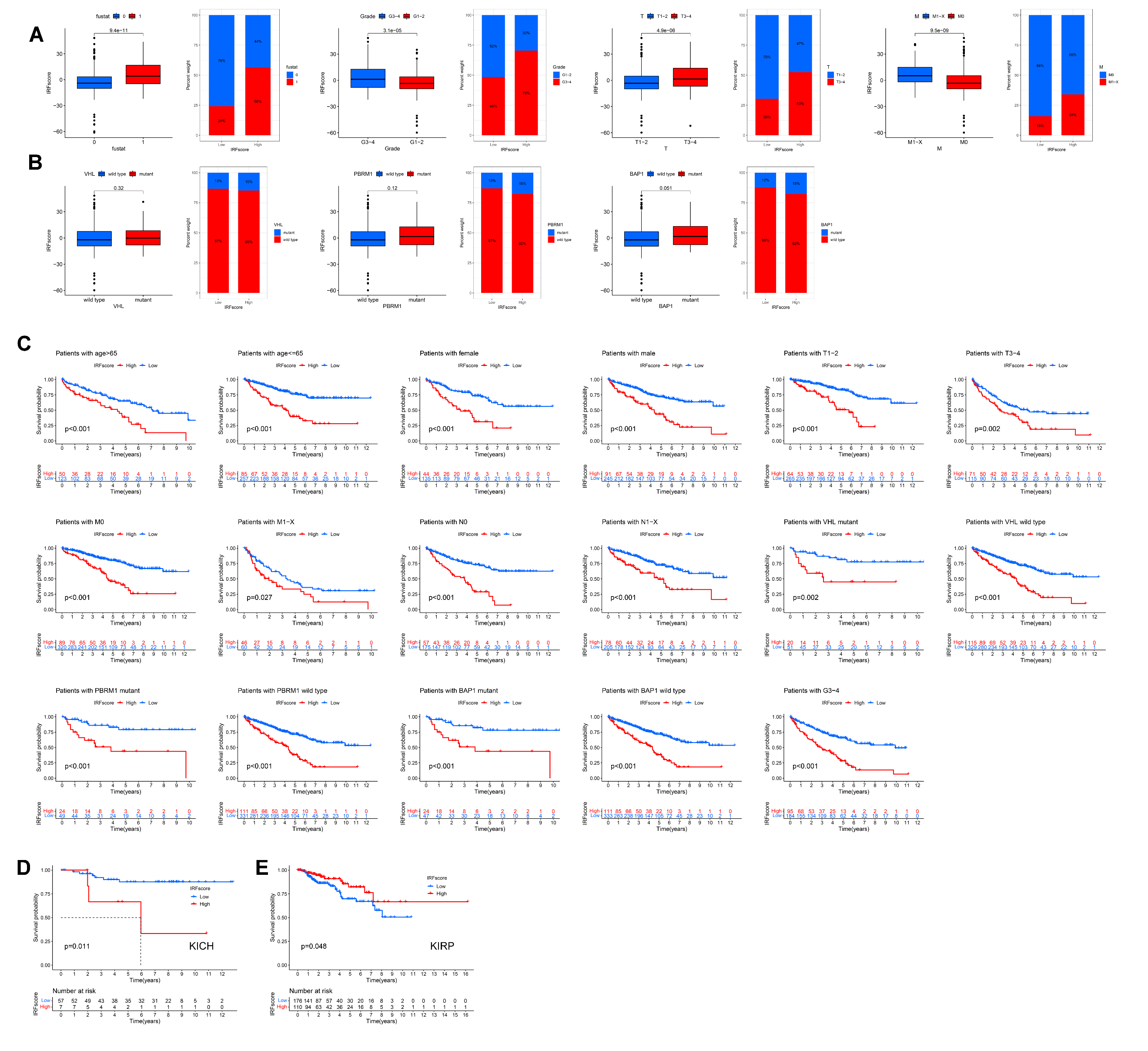


**Figure S5** clinical characteristics of IRFscore in OC patient subtypes and other two kidney tumour. **(A)** Boxplots and the proportion for IRFscore between different characteristics ccRCC patients, including patient survival status, grade, T and M. **(B)** Boxplots and the proportion for IRFscore between genetic mutations including VHL, PBRM1 and BAP1 mutations. **(C)** Kaplan-Meier curves depicted the survival difference between low and high IRFscore in the stratified analysis of OC patients. **(D)** Kaplan-Meier curves depicted the survival difference between low and high IRFscore in KICH. **(E)** Kaplan-Meier curves depicted the survival difference between low and high IRFscore in KIRP.


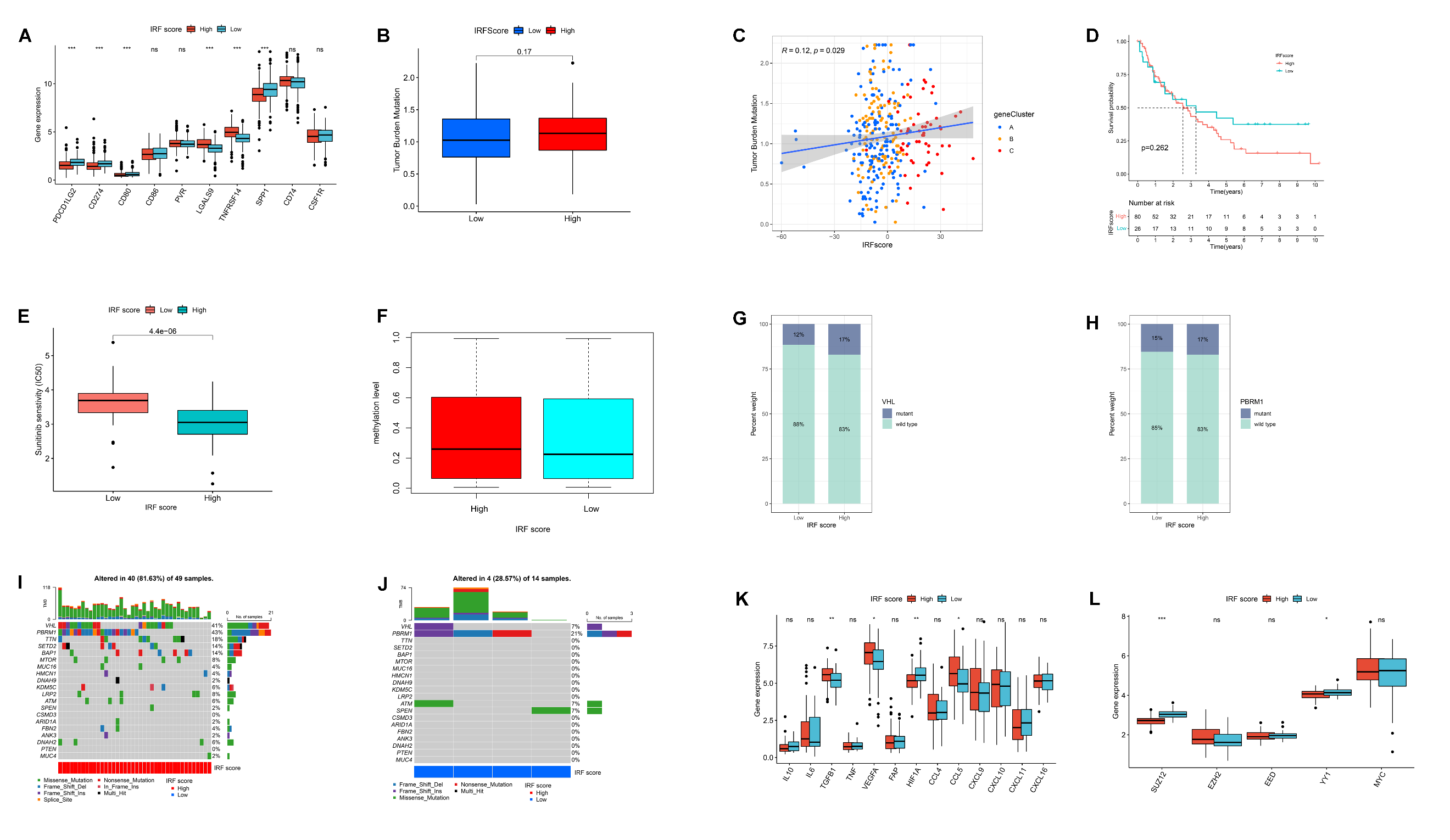


**Figure S6** IRF-related molecular subtypes in the ccRCC immunological and molecular subtypes. **(A)** The differences in the receptors or ligands expressed by M2 macrophages between the high and low IRFscore groups. **(B)** TMB difference in the high and low IRFscore groups. **(C)** Scatterplots depicting the positive correlation between IRFscores and TMB. **(D)** Kaplan-Meier survival analysis for the high and low IRFscore groups in mccRCC cohort. **(E)** Box plot showing the sensitivity of mccRCC patients with high and low IRFscore subgroups to sunitinib. **(F)** The differences in methylation levels between the high and low IRFscore groups in mccRCC cohort. **(G)** The proportion of VHL mutations in high and low IRFscore groups in mccRCC cohort. **(H)** The proportion of PBRM1 mutations in high and low IRFscore groups in mccRCC cohort. **(I)** The single-nucleotide variant was constructed using high IRFscore in mccRCC cohort. **(J)** The single-nucleotide variant was constructed using low IRFscore in mccRCC cohort. **(K)** The differences in inflammatory factors between the high and low IRFscore groups in mccRCC cohort. **(L)** The differences in stem cell differentiation markers between the high and low IRFscore groups in mccRCC cohort.
